# Development and validation of a *PBRM1*‐associated immune prognostic model for clear cell renal cell carcinoma

**DOI:** 10.1002/cam4.4115

**Published:** 2021-09-18

**Authors:** Jiayi Chen, Chunlin Yao, Nan Qiao, Yangyang Ge, Jianhua Li, Yun Lin, Shanglong Yao

**Affiliations:** ^1^ Department of Anesthesiology Institute of Anesthesia and Critical Care Medicine Union Hospital Tongji Medical College Huazhong University of Science and Technology Wuhan China

**Keywords:** clear cell renal cell carcinoma, immune prediction model, mutation, PBRM1, tumor immunology

## Abstract

Alteration in the polybromo‐1 (*PBRM1*) protein encoding gene *PBRM1* is the second most frequent mutation in clear cell renal cell carcinoma (ccRCC). It causes a series of changes in the tumorigenesis, progression, prognosis, and immune response of ccRCC patients. This study explored the *PBRM1*‐associated immunological features and identified the immune‐related genes (IRGs) linked to *PBRM1* mutation using bioinformatics methods. A total of 37 survival IRGs associated with *PBRM1* mutation in ccRCC patients were identified. To further explore the role of these IRGs in ccRCC and their association with immune status, eight IRGs with remarkable potential as individual targets were selected. An immune model that was constructed showed good performance in stratifying patients into different subgroups, showing clinical application potential compared to traditional clinical factors. Patients in the high‐risk group were inclined to have more advanced stage and higher grade tumors with node metastasis, distant metastasis, and poorer prognosis. Furthermore, these patients had high percentages of regulatory T cells, follicular helper T cells, and M0 macrophages and exhibited high expression levels of immune checkpoints proteins, such as CTLA‐4, PD‐1, LAG‐3, TIGIT, and CD47. Finally, a nomogram integrating the model and clinical factors for clinical application showed a more robust predictive performance for prognosis. The prediction model associated with *PBRM1* mutation status and immunity can serve as a promising tool to stratify patients depending upon their immune status, thus facilitating immunotherapy in the future.

## Graphical abstract

In this study, we used bioinformatic methods to explore the effect of PBRM1 mutation in clear cell renal cell carcinoma samples on the immune status and identify immune‐related biomarkers related to PBRM1 mutation. As far as we know, this is the first report of a prediction model incorporating PBRM1 status and it serves as a promising tool to determine the immune status and stratify patients to judge the effectiveness of immunotherapy.

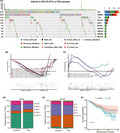

## 1 INTRODUCTION

Renal cell carcinoma (RCC), a frequently diagnosed urological tumor, accounts for about 2%–3% of all malignant tumors.^1^ Clear cell renal cell carcinoma (ccRCC) is the most common histological subtype with high mortality rate when metastasized.^2^ Development of effective treatment regimen for prolonged survival of advanced ccRCC patients is still in its initial stage, owing to the lack of theoretical understanding of the molecular pathways underlying ccRCC carcinogenesis. The immunotherapeutic revolution currently underway in medical oncology provides alternative therapeutic strategies for advanced ccRCC patients.^3^ Several clinical studies have suggested that immune checkpoint inhibitors could improve the survival of advanced ccRCC patients by enhancing the immune function and mediating the antitumor activity.^4^, ^5^ In addition, many immune‐related indicators have been proposed to evaluate the outcome of ccRCC,^6^, ^7^ thus emphasizing on the importance of the immune microenvironment. However, tumor immune microenvironment in ccRCC and its association with patient prognosis and response to immunotherapy have not been investigated comprehensively.

Alteration in polybromo‐1 (*PBRM1*) protein encoding gene *PBRM1* is the second most frequent mutation in ccRCC and is found in nearly 40% of the ccRCC tissues^8^ and in all or majority of the cancer cells of ccRCC.^9^ *PBRM1* encodes BAF180 protein, which serves as a subunit of the polybromo BAF switch‐sucrose non‐fermentable (SWI/SNF) complex. The SWI/SNF chromatin remodeling complexes carry out various cellular functions, including cell differentiation, programmed cell death, cell cycle, cell metabolism, genomic instability, and DNA repair,^10^ and its loss of function remains associated with malignant transformation.^11^ Previous studies have suggested that *PBRM1* knockdown promotes proliferation and invasion of ccRCC tumor cells.^8^ However, the underlying mechanism of how *PBRM1* mutation promotes tumorigenesis and tumor progression remains unclear. Recently, a study by Miao et al. demonstrated that *PBRM1* mutations in ccRCC might remodel the tumor immune microenvironment to improve the clinical responses to immune checkpoint inhibitors.^12^ Pan et al. demonstrated that tumor cells with *PBRM1* mutation enhance the sensitivity to T‐cell‐mediated cytotoxicity and produce higher amounts of chemokines. These chemokines then recruit a greater number of effector T cells into the tumors, which enhances the response of ccRCC patients to immunotherapy.^13^ Despite the extensive development of high‐throughput sequencing of cancer genomes, key candidates activated by *PBRM1* in cytokine production and inflammation pathways are not known. Thus, identification of the candidate biomarkers related to *PBRM1* status and understanding of the exact effects of *PBRM1* on the pathogenesis of ccRCC are critical.

In this study, *PBRM1* mutation status combined with RNA expression data and clinical phenotype were systematically analyzed to explore the effects of *PBRM1* mutation on the immune status and *PBRM1*‐associated immune microenvironment in ccRCC patients.

## 2 MATERIALS AND METHODS

### 2.1 Data collection

**FIGURE 1:**
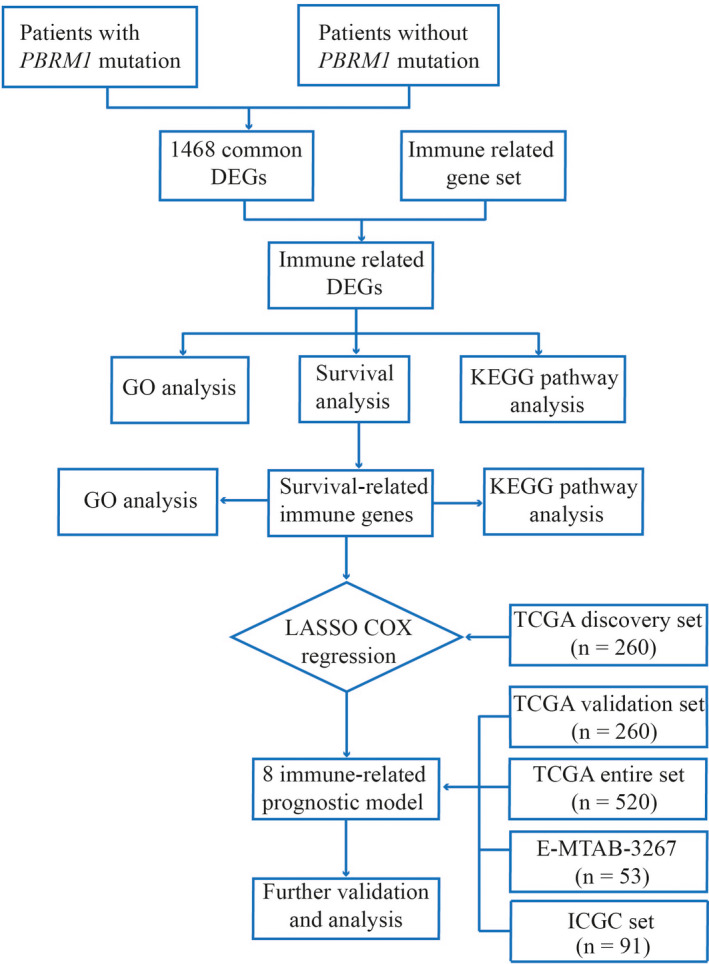
Flow chart illustrating the process of the study

The RNA‐sequencing data and the corresponding clinical dataset for 530 ccRCC patients were downloaded from the Cancer Genome Atlas (TCGA) data portal (https://portal.gdc.cancer.gov/repository) in August 2019. The somatic mutation statuses of 336 ccRCC patients (identified by VarScan2) were also obtained from the TCGA database. Among these ccRCC patients, 332 with *PBRM1* status were screened for differentially expressed genes (DEGs). A file of immune‐related genes (IRGs) was downloaded from the Immunology Database and Analysis Portal (ImmPort; https://immport.niaid.nih.gov),^14^ containing a total of 1534 human IRGs (Table S1). These genes from ImmPort have been identified to be involved with the immune response. Another dataset, E‐MTAB‐3267, containing 53 tumor tissue samples, was obtained from the ArrayExpress database (https://www.ebi.ac.uk/arrayexpress/). We also downloaded a dataset (number as RECA‐EU) from the International Cancer Genomics Consortium (ICGC) database (https://dcc.icgc.org/), containing 91 tumor tissue samples. These two datasets were used for external validation. The workflow of this study is demonstrated in Figure 1.

### 2.2 Data preprocessing

For the RNA‐sequencing data obtained from the TCGA dataset, Homo_sapiens.GRCh38.97.chr.gtf file was used to annotate the gene symbols. Data normalization was implemented as follows: (1) Raw data were normalized using the trimmed mean of *M*‐values method with the edgeR package in R software (Version 3.5.3; https://www.r‐project.org/). (2) The duplicate genes were averaged. (3) Genes with low expression value (an average expression value < 1) were removed. (4) The expression of all gene symbols was log2 (normalized value + 1) transformed. For the E‐MTAB‐3267 microarray data, robust multi‐array averaging method was used for background correction and these probes were annotated using corresponding Affymetrix annotation files. To investigate the tumor‐associated immune microenvironment of these ccRCC patients, we used the CIBERSORT algorithm (https://cibersort.stanford.edu) to infer the proportions of 22 immune cell types.^15^ The RNA‐sequencing data from the TCGA database for the CIBERSORT algorithm were processed following the steps mentioned below. The RNA‐sequencing data were transformed to normalized gene expression data using the “voom” method, converting count data to a microarray similar result.^16^ The mRNA expression profiles were then normalized using the “limma” package and the data for duplicate genes were averaged. Normalized gene expression data were retained for subsequent analysis.

### 2.3 Gene set enrichment analysis

To determine the biological pathways associated with *PBRM1* mutation and immunity in the TCGA ccRCC cohort, gene set enrichment analysis (GSEA, http://software.broadinstitute.org/gsea/index.jsp) was performed involving the *PBRM1* mutated (*PBRM1* ^MUT^) (*n* = 135) and *PBRM1* wild‐type (*PBRM1* ^WT^) (*n* = 197) ccRCC patients. The c5.bp.v7.0.symbols.gmtfile was selected as the reference file. *p*‐value < 0.05 was set as the cutoff criterion.

### 2.4 Identification of the differentially expressed IRGs

To obtain the DEGs associated with *PBRM1* status, differential expression analysis involving *PBRM1* ^MUT^ (*n* = 135) and *PBRM1* ^WT^ (*n* = 197) ccRCC patients was performed using the “edgeR” package.^17^ The false discovery rate (FDR) < 0.05 and log2 |fold change| > 1 were selected as the thresholds. To obtain differentially expressed IRGs associated with *PBRM1* mutation, DEGs associated with *PBRM1* mutation were mapped on the gene list available from the ImmPort website.

### 2.5 Development and validation of an immune‐related prediction model

Among the 530 ccRCC patients with survival information, 10 patients with <1 week of follow‐up were excluded. A total of 520 ccRCC patients were randomly assigned to the discovery (*n* = 260) and validation (*n* = 260) cohorts. The discovery cohort was used to develop an immune‐related prediction model. Validation and total cohorts were used for internal validation. The E‐MTAB‐3267 and ICGC‐RECA datasets externally validated the performance of the prediction model. The detailed characteristics of all cohorts are presented in Table 1. In the discovery cohort, univariate Cox regression analysis was performed using the “survival” R package to select survive‐associated IRGs. *p*‐value < 0.05 was set as the threshold criterion. To minimize overfitting, least absolute shrinkage and selection operator (LASSO) analysis was conducted with “glmnet” package to select the most valuable genes for predicting the survival of the ccRCC patients.^18^ The 10‐fold cross‐validation was used to determine the optimal value of the penalty parameter and a sub‐group of IRGs involved in ccRCC patient prognosis was selected. We developed an immune‐related prediction model based on the regression coefficients of LASSO analysis weighted with the expression of these selected genes.^19^, ^20^ According to the median risk score value, patients were then classified into high‐ and low‐risk subgroups.

**TABLE 1:** Clinical characteristics of the clear cell renal cell carcinoma patients in each dataset

Variables	Discovery set (*n* = 260), *n* (%)	Validation set (*n* = 260), *n* (%)	*p* value	Entire set (*n* = 520), *n* (%)	E‐MTAB‐3267 *n* (%)	ICGC *n* (%)
Age (mean ± SD, years)	60.2 ± 12.2	60.4 ± 11.9	0.830	60.3 ± 12.0	59.8 ± 8.2	60.5 ± 10.0
≤60 years	127 (48.8)	136 (52.3)	0.483	263 (50.6)	31 (58.5)	46 (50.5)
>60 years	133 (51.2)	124 (47.7)		257 (49.4)	22 (41.5)	45 (49.5)
Gender			1			
Male	171 (65.8)	171 (65.8)		342 (65.8)	37 (69.8)	52 (57.1)
Female	89 (34.2)	89 (34.2)		178 (34.2)	16 (30.2)	39 (42.9)
Stage			0.010			
I	130 (50.0)	129 (49.6)		259 (49.8)	NA	NA
II	22 (8.5)	33 (12.7)		55 (10.6)	NA	NA
III	69 (26.5)	53 (20.4)		122 (23.4)	NA	NA
IV	37 (14.2)	44 (16.9)		81 (15.6)	NA	NA
Unknown	2 (0.8)	1 (0.4)		3 (0.6)	NA	NA
Grade			0.755			
G1	6(2.3)	6 (2.3)		12 (2.3)	NA	NA
G2	116 (44.6)	108 (41.5)		224 (43.1)	NA	NA
G3	103 (39.6)	100 (38.5)		203 (39.1)	NA	NA
G4	32 (12.3)	41 (15.8)		73 (14.0)	NA	NA
Unknown	3 (1.2)	5 (1.9)		8 (1.5)	NA	NA
p_T			0.136			
T1	134 (51.6)	131 (50.4)		265 (51.0)	NA	51 (56.0)
T2	25 (9.6)	42 (16.2)		67 (12.9)	NA	9 (9.9)
T3	95 (36.5)	82 (31.5)		177 (34.0)	NA	27 (29.7)
T4	6 (2.3)	5 (1.9)		11 (2.1)	NA	1 (1.1)
Unknown	0 (0)	0 (0)		0 (0)	NA	3 (3.3)
p_M			0.782			
M0/Mx	224 (86.2)	218 (83.8)		442 (85.0)	NA	87 (95.6)
M1	36 (13.8)	42 (16.2)		78 (15.0)	NA	4 (4.4)
p_N			0.683			
N0/Nx	254 (97.7)	250 (96.2)		504 (96.9)	NA	41 (45.1)
N1	6 (2.3)	10 (3.8)		16 (3.1)	NA	50 (54.9)
Status			0.450			
Dead	77 (29.6)	86 (33.1)		163 (31.3)	39^a^ (73.6)	30 (33.0)
Alive	183 (70.4)	174 (66.9)		357 (68.7)	14^b^ (26.4)	61 (67.0)

Abbreviations: ICGC, International Cancer Genomics Consortium; NA, not avaliable; SD, standard deviation.

^a^ Progression.

^b^ No‐progression.

To evaluate the sensitivity and specificity of the prediction model, time‐dependent receiver operating characteristic (ROC) analysis was performed, and the areas under the curve were calculated using the Kaplan–Meier (K–M) “survival ROC” R package.^21^ Pearson’s correlation analysis evaluated the relationship between risk score and survival time. K–M analysis and log‐rank test for prognostic evaluation were performed using the “survminer” R package. The predictive ability was estimated using the Harrell's concordance index (*C*‐index).

### 2.6 Immunohistochemistry

Fresh tissue samples of ccRCC and matched normal samples were collected at Union hospital, Tongji Medical college, Huazhomorng University of Science and Technology in May 2021. The pathology results of ccRCC were confirmed by two pathologists, respectively. Informed consents were acquired from the concerned patients, and research were approved by the Ethics Committee of Union hospital, Tongji Medical college, Huazhong University of Science and Technology. These samples were fixed with polyformaldehyde, and the expressions of FGF23 and PTGER1 in ccRCC and matched normal samples were confirmed using immunohistochemistry (IHC). The primary antibodies used were: anti‐FGF23 (DF3596; 1:100; Affinity) and anti‐PTGER1 (DF10213; 1:200; Affinity). The staining of FGF23 and PTGER1 was operated using a standard 3,3′‐diaminobenzidine protocol. Images of FGF23 and PTGER1 were captured using a Leica microscope.

### 2.7 Functional and pathway enrichment analysis

The Database for Annotation, Visualization, and Integrated Discovery (https://david.ncifcrc.gov/) ^22^ was used to perform Gene Ontology and Kyoto Encyclopedia of Genes and Genomes (KEGG) pathway enrichment analysis for these differentially expressed IRGs associated with *PBRM1* mutation and survive‐associated IRGs. FDR < 0.01 was set as the threshold criterion.

### 2.8 Estimation of the degree and types of infiltrating immune cells

Stromal and immune cells are the two main non‐tumor components in the tumor microenvironment, which have been proposed to be valuable in the treatment and prognostic assessment of tumors. To investigate the tumor microenvironment of different risk groups, immune and stromal scores for the total TCGA cohorts reflecting the infiltration levels of non‐tumor cells were calculated using the ESTIMATE package.^23^ Differences in the immune and stromal scores of ccRCC were compared for the low‐ and high‐risk groups. Normalized RNA‐sequencing data from TCGA database combined with LM22 signature matrix were used for the CIBERSORT algorithm to evaluate the relative proportion of 22 types of immune cells in each risk group as previously described.^24^ Then, we compared the immune cell phenotypes of the low‐ and high‐risk groups.

### 2.9 Independence analysis of the prediction model based on the clinical factors

We used the total TCGA cohorts to perform the independence analysis. Among the included ccRCC patients, 509 patients with complete information of clinical factors, including age, sex, pathological stage, histological grade, pathological T staging, pathological N staging, and pathological M staging, were selected. Univariate and multivariate Cox regression analyses were performed to screen the independent prognostic indicators.

### 2.10 Establishment and assessment of nomogram

Four independent prognostic indicators were used to establish a nomogram for better prediction of prognosis using the “rms” R package.^25^ The “top points” are assigned for age, grade, stage, and risk score by drawing a line upward from the corresponding values to the “top points.” The bottom “total points” line represents the sum of these points and corresponds to the predictions of 1‐, 3‐, and 5‐year overall survival. Next, the accuracy of the nomogram for predicting patient survival (1‐, 3‐, and 5‐year) was estimated using calibration curves. In addition, the *C*‐index was calculated and compared to evaluate the predictive accuracy of the nomogram. For the external validation of the nomogram, the total points for each patient in the validation dataset were calculated according to the established nomogram; Cox regression in the validation dataset was performed using the total points as a factor; and the calibration curves were derived based on the regression analysis.

## 3 RESULTS

### 3.1 Relationship between immune status and *PBRM1* mutation in ccRCC

**FIGURE 2:**
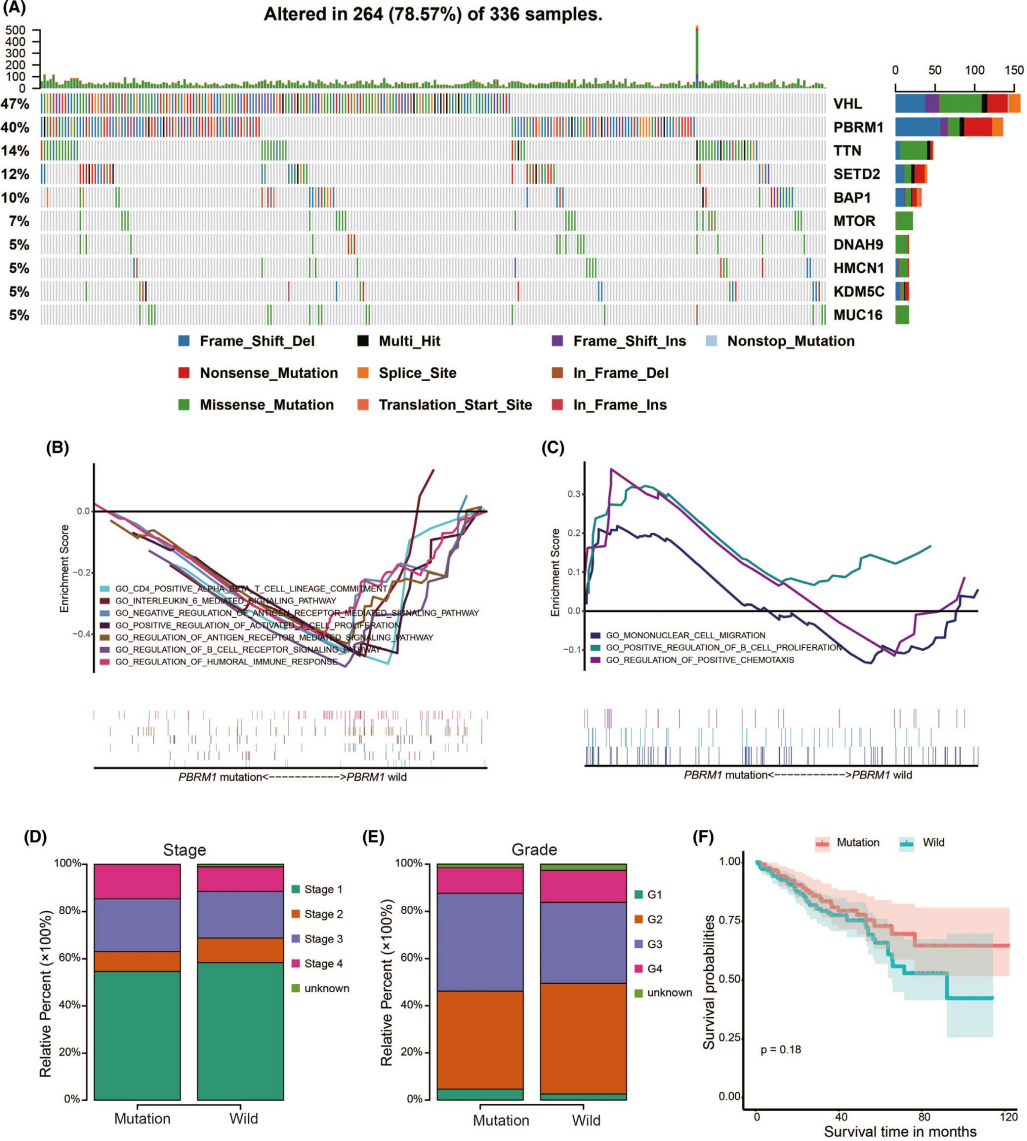
Correlation between *PBRM1* mutation and clinicopathological factors of ccRCC. (A) Mutation landscape of top 10 genes in ccRCC in the Cancer Genome Atlas dataset. (B,C) The immune‐related biological processes significantly enriched in *PBRM1* wild‐type (*PBRM1* ^WT^) and *PBRM1* mutated (*PBRM1* ^MUT^) ccRCC patients. Relative proportions of immune cells for pathological stage (D) and grade (E) of ccRCC patients with and without *PBRM1* mutation. (F) Survival curve showing no significant difference between ccRCC patients with and without *PBRM1* mutation. ccRCC, clear cell renal cell carcinoma

In ccRCC, *PBRM1* mutation is the second most frequently mutated gene, occurring in about 40% of the ccRCC tumors (Figure 2A). On comparing the immune status of *PBRM1* ^MUT^ (*n* = 135) and *PBRM1* ^WT^ ccRCC patients, GSEA (*n* = 197) showed that *PBRM1* ^WT^ ccRCC patients were highly enriched in 278 biological processes, of which 7 were immune‐related. The immune‐related biological processes include regulation of B‐cell receptor signaling pathway (normalized enrichment score [NES] = −1.534, size = 27), regulation of humoral immune response (NES = −1.395, size = 67), CD4 positive alpha–beta T‐cell lineage commitment (NES = −1.483, size = 17), positive regulation of activated T‐cell proliferation (NES = −1.457, size = 24), interleukin 6‐mediated signaling pathway (NES = −1.473, size = 19), negative regulation of antigen receptor‐mediated signaling pathway (NES = −1.451, size = 27), and regulation of antigen receptor‐mediated signaling pathway (NES = −1.459, size = 61; Figure 2B; Table S2). In contrast, *PBRM1* ^MUT^ ccRCC patients were enriched in three immune‐related biological processes, namely regulation of positive chemotaxis (NES = 1.493, size = 26), positive regulation of B‐cell proliferation (NES = 1.485, size = 39), and mononuclear cell migration (NES = 1.164, size = 82; Figure 2C; Table S3). These findings indicate that immune status varies between the *PBRM1* ^MUT^ and *PBRM1* ^WT^ ccRCC patients. There is a discrepancy in the association between loss of *PBRM1* and tumor grade.^26^ We then analyzed the relative proportions for pathological stage and grade between the ccRCC patients with and without *PBRM1* mutation (Figure 2D,E). The ccRCC patients with *PBRM1* mutation were inclined to have relatively high proportion of advanced stage and high‐grade tumors. The K–M survival curve showed no significant difference in the survival of ccRCC patients with and without *PBRM1* mutation (Figure 2F).

### 3.2 Identification of the IRGs expression differences between *PBRM1* ^MUT^ and *PBRM1* ^WT^ ccRCC patients

**FIGURE 3:**
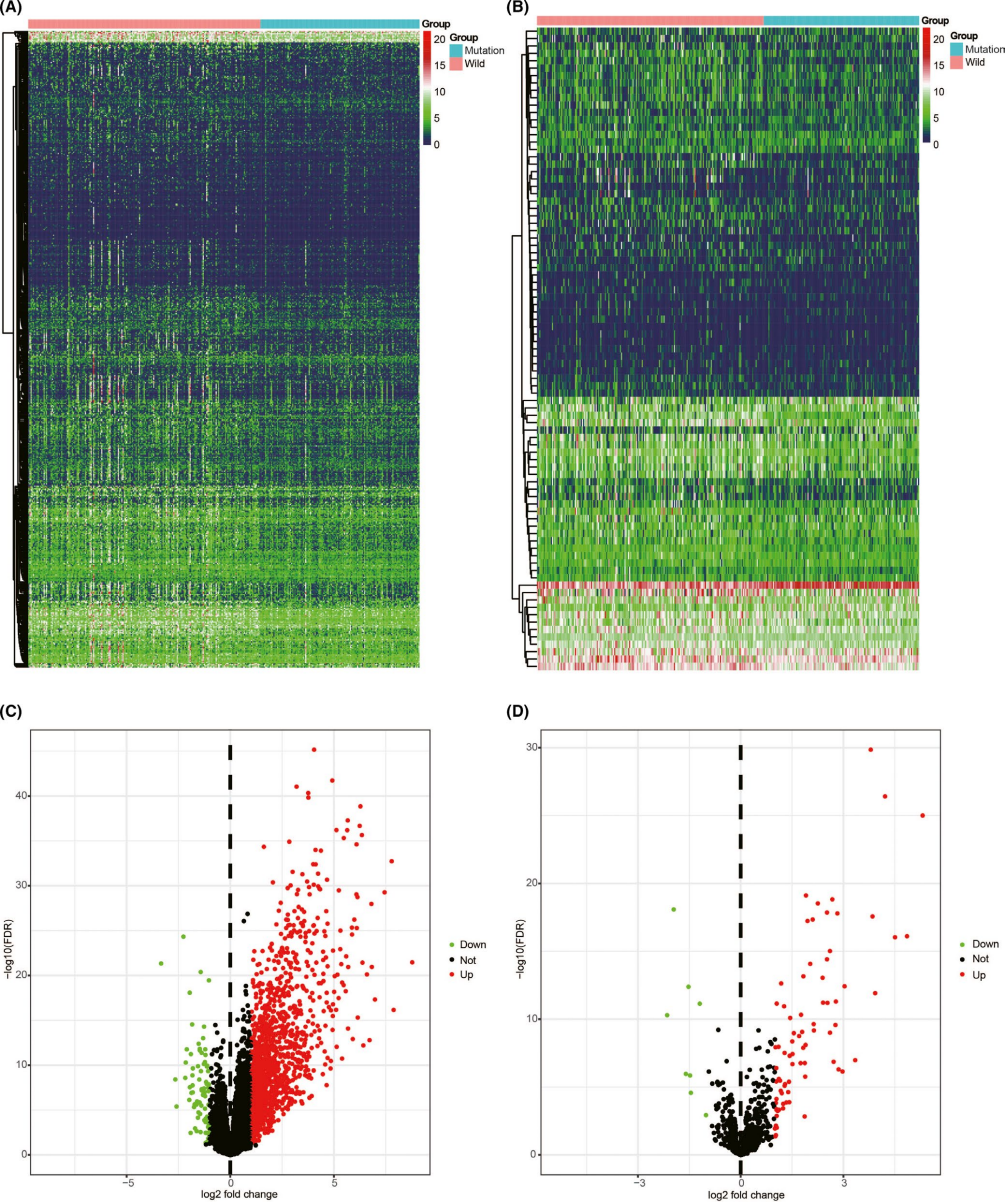
Differentially expressed IRGs. A heatmap (A) and a volcano plot (C) showing differentially expressed genes between *PBRM1* wild‐type (*PBRM1* ^WT^) and *PBRM1* mutated (*PBRM1* ^MUT^) ccRCC patients. Differentially expressed IRGs are shown in a heatmap (B) and a volcano plot (D). The green dots represent downregulated genes, the red dots represent upregulated genes, and the black dots represent genes which were not differentially expressed. IRG, immune‐related gene

**FIGURE 4:**
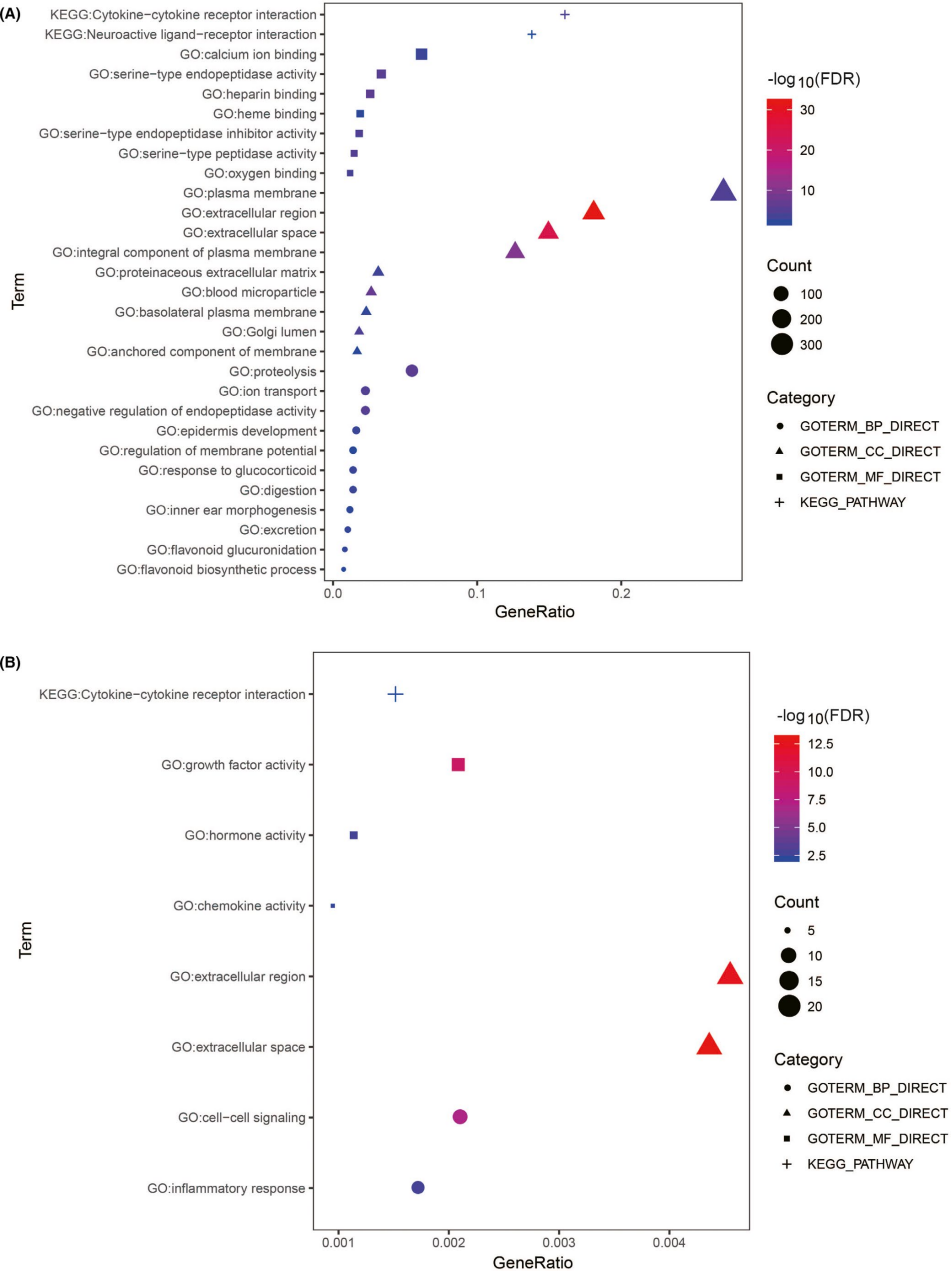
Functional enrichment analysis. The results of Gene Ontology and Kyoto Encyclopedia of Genes and Genomes pathway analysis based on 87 differentially expressed IRGs (A) and 37 survive‐associated IRGs (B). IRG, immune‐related gene

To identify the candidate biomarkers related to *PBRM1* status, differential expression analysis was performed with *PBRM1* ^WT^ and *PBRM1* ^MUT^ ccRCC tissues using the “edgeR” package.^17^ Out of the total 1468 genes identified, 1388 and 80 genes were upregulated and downregulated, respectively (Figure 3A,C). The inflammatory molecules, including chemokines, cytokines, and their receptors expressions were compared between the *PBRM1* ^WT^ and *PBRM1* ^MUT^ ccRCC patients (Figure S1A). In addition, the expression differences of major histocompatibility complex molecules, co‐stimulators, and co‐inhibitors were also compared between the *PBRM1* ^WT^ and *PBRM1* ^MUT^ ccRCC patients (Figure S1B). These results suggest the presence of different immune and inflammatory states between the *PBRM1* ^WT^ and *PBRM1* ^MUT^ ccRCC patients. Results of gene functional enrichment analysis for the DEGs in *PBRM1* ^WT^ and *PBRM1* ^MUT^ ccRCC patients are shown in Figure S1C. To explore the biomarkers associated with immune status, a list of 1534 human IRGs was obtained from ImmPort. DEGs associated with *PBRM1* mutation were mapped to the set of human IRGs; a total of 87 different IRGs were identified, including 79 upregulated and 8 downregulated genes (Figure 3B,D). Detailed information is shown in Table S4. Gene functional enrichment analysis based on four differentially expressed IRGs revealed that the most significant biological process, cellular component, and molecular function were “proteolysis,” “extracellular region,” and “heparin binding,” respectively (Figure 4A). The most significantly enriched KEGG pathway was “cytokine–cytokine receptor interaction” (Figure 4A), indicating the role of these candidate biomarkers in the immune response.

### 3.3 Identification of survive‐associated IRGs

In the discovery cohort, univariate Cox regression analysis was performed to select the survive‐associated IRGs, revealed 37 IRGs to be significantly associated with survival in ccRCC patients (*p* < 0.05). Gene functional enrichment analysis based on the survive‐associated IRGs revealed that the most significant biological process, cellular component, and molecular function were “cell–cell signaling,” “extracellular space,” and “growth factor activity,” respectively (Figure 4B). KEGG analysis revealed that the most significantly enriched pathway was “cytokine–cytokine receptor interaction” (Figure 4B).

### 3.4 Construction of the immune‐related prediction model in the discovery cohorts

**FIGURE 5:**
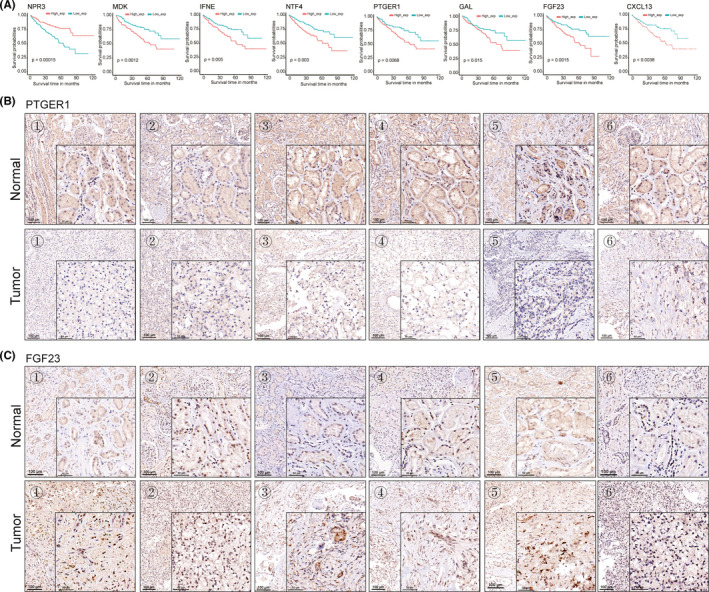
Survival analysis of eight immune‐related genes used to construct the immune prediction model for ccRCC in the Cancer Genome Atlas discovery cohorts and immunohistochemistry verification. (A) Kaplan–Meier survival analysis for genes, comprising *NPR3*, *MDK*, *IFNE*, *NTF4*, *PTGER1*, *GAL*, *FGF23*, and *CXCL13*. The results of immunohistochemistry showed low expressions of *PTGER1* (B) and high expressions of *FGF23* (C) in ccRCC compared with matched normal tissues. The same enclosed numbers represent the tumor and adjacent normal tissues of the same patient for *PTGER1* and *FGF23*, respectively. ccRCC, clear cell renal cell carcinoma

Considering the differences in the immune phenotype and gene expression between *PBRM1* ^WT^ and *PBRM1* ^MUT^ccRCC patients, we evaluated the predictive ability of the IRGs in combination. LASSO analysis was used to screen the most valuable genes for predicting prognosis in TCGA discovery cohort (Figure S2). Finally, eight most valuable prognostic IRGs comprises of natriuretic peptide receptor 3 (*NPR3*), midkine (*MDK*), interferon epsilon (*IFNE*), neurotrophin‐4 (*NTF4*), prostaglandin E2 receptor EP1 subtype (*PTGER1*), galanin peptides (*GAL*), fibroblast growth factor 23 (*FGF23*), and C‐X‐C motif chemokine ligand 13 (*CXCL13*). Detailed information is shown in Table S5. In *PBRM1* ^MUT^ samples, *MDK*, *IFNE*, *NTF4*, *PTGER1*, *GAL*, *FGF23*, and *CXCL13* were upregulated and *NPR3* was downregulated. The K–M curves showed that all the eight genes were correlated with patient survival (*p* < 0.05, Figure 5A). Moreover, the TIMER database was used to evaluate the correlation of the expression level of these genes with immune cells (https://cistrome.shinyapps.io/timer/).^27^ The results shown in Figure S3 indicated different immune statuses of these genes. In addition, we found that PTGER1 was low‐expressed (Figure 5B), and FGF23 was high‐expressed (Figure 5C) in ccRCC compared with matched normal tissues using IHC. Based on the results of the LASSO analysis, we established a prediction model for ccRCC patients using the expression levels of the genes as follows: Risk score = (−0.113150947 * *NPR3*) + (0.046765504 * *MDK*) + (0.024601593 * *IFNE*) + (0.039980648 * *NTF4*) + (0.011819219 * *PTGER1*) + (0.036773937 * *GAL*) + (0.020128759 * *FGF23*) + (0.006482745 * *CXCL13*). The *C*‐index for TCGA discovery cohort was 0.703. Patients were classified into high‐ (*n* = 130) and low‐risk (*n* = 130) groups based on their median risk score (0.972). High‐risk patients showed poor prognosis compared to the low‐risk patients (Figure S4A). The risk score of the prediction model was inversely related with the survival time (Figure S4B). ROC analysis suggested that the prediction accuracy of the model was 0.739 at 0.5 year, 0.719 at 1 year, 0.658 at 2 years, 0.708 at 3 years, and 0.768 at 5 years (Figure S4C). The K–M curve indicated that patients in the high‐risk group had worse survival than those in the low‐risk group (*p* < 0.05, Figure S4D).

### 3.5 Validation of the immune‐related prediction model

To determine the prognostic value of the *PBRM1*‐associated model, we applied the same formula in TCGA validation cohort, TCGA total cohort, and E‐MTAB‐3267 and ICGC‐RECA cohorts to calculate the risk score for each patient. Using the same cutoff value (0.972) from the discovery cohort, each patient in the validation and total cohorts were marked as high‐ or low‐risk. The ccRCC patients in E‐MTAB‐3267 and ICGC‐RECA cohorts were classified into high‐ and low‐risk groups based on their median risk score (0.212 and 0.100, respectively). The *C*‐indexes indicated favorable predictive accuracy of the prediction model in the validation (*C*‐indexes: 0.694), total (*C*‐indexes: 0.688), E‐MTAB‐3267 (*C*‐indexes: 0.731), and ICGC‐RECA (*C*‐indexes: 0.592) cohorts. In all the cohorts, the patients of the high‐risk group had more tumor‐associated deaths than those belonging to the low‐risk group (Figure S4E,I,M,Q). The risk scores of the model in the validation, total, E‐MTAB‐3267, and ICGC‐RECA cohorts were also negatively correlated with the survival time (Figure S4F,J,N,R). The ROC curves of the prediction model in validation, total, E‐MTAB‐3267, and ICGC‐RECA cohorts had high sensitivity and specificity, suggesting good prognostic accuracy of the model for ccRCC patients (Figure S4G,K,O,S). In the validation, total, and E‐MTAB‐3267 cohorts, survival analysis showed that ccRCC patients in the high‐risk group had poorer prognosis than those in the low‐risk group (Figure S4H,L,P). In ICGC‐RECA cohort, high‐risk patients tended to have worse prognosis compared to the low‐risk patients, despite *p* > 0.05 (Figure S4T).

### 3.6 Stratification analyses and association with clinicopathological factors

**FIGURE 6:**
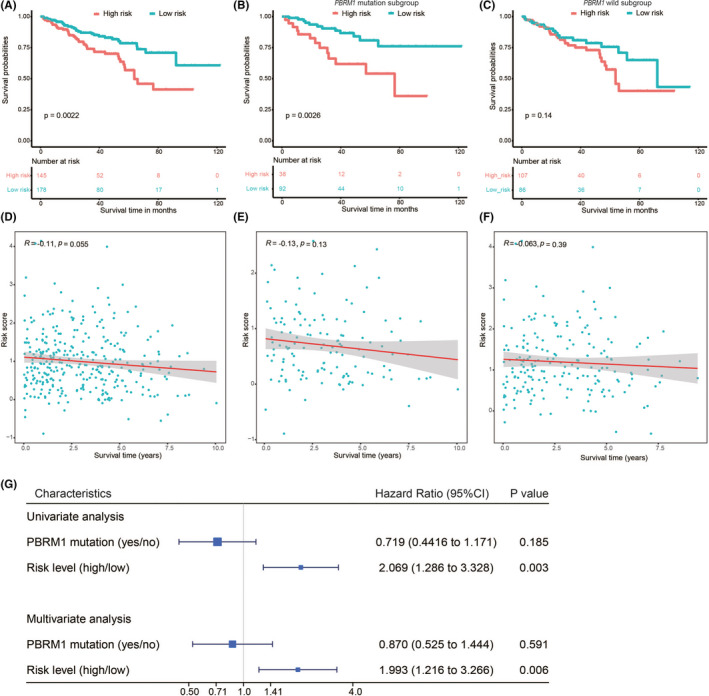
Prognostic analysis of the *PBRM1* mutation. Kaplan–Meier survival analysis of ccRCC patients with *PBRM1* status (A), *PBRM1* mutation (*PBRM1* ^MUT^) (B), and *PBRM1* wild‐type (*PBRM1* ^WT^) subgroup (C). Correlation between the survival time and the risk score for ccRCC patients with *PBRM1* status (D), *PBRM1* ^MUT^ (E), and *PBRM1* ^WT^ subgroups (F). (G) Univariate and multivariate Cox regression analyses between the model and *PBRM1* status regarding the prognostic value. ccRCC, clear cell renal cell carcinoma

Consistent with the results of the immune‐related prediction model for all ccRCC patients, patients with *PBRM1* status data also showed that high‐risk patients tended to have poor prognosis compared to the low‐risk patients (Figure 6A). Stratification analyses also suggested that poor prognosis remains associated with the high‐risk patients for both *PBRM1* ^MUT^ (Figure 6B) and *PBRM1* ^WT^ TCGA ccRCC cohorts (Figure 6C). Moreover, risk score of the immune‐related prediction model was negatively correlated with the survival time in ccRCC patients with different *PBRM1* status (Figure 6D), *PBRM1* ^MUT^ (Figure 6E), and *PBRM1* ^WT^ TCGA cohorts (Figure 6F). Univariate and multivariate Cox regression analyses indicated that the immune‐related prediction model for the overall survival of ccRCC patients is independent of *PBRM1* mutation status (Figure 6G). The relationship between clinical factors and risk score of the immune‐related prediction model is shown in Figure S5. We found that male, high‐grade, advanced stage, high T stage, node metastasis, distant metastasis, and high‐risk patients were inclined to have a score that represents high risk (*p* < 0.05).

### 3.7 High‐risk ccRCC patients indicated an enhanced local immune phenotype

**FIGURE 7:**
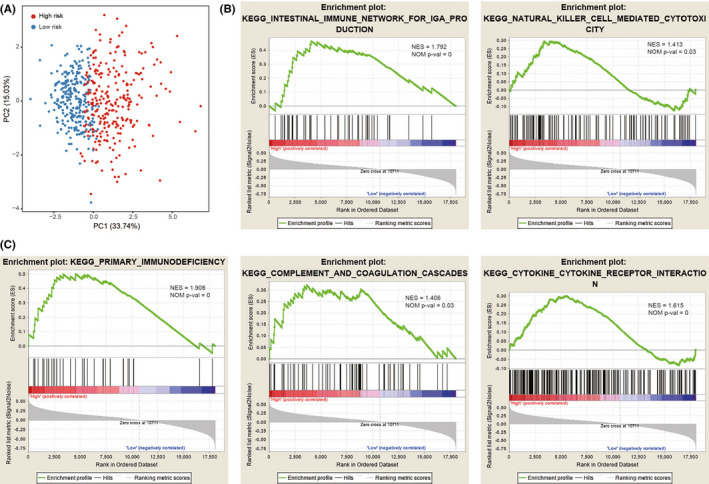
Enhanced local immune phenotype in high‐risk ccRCC patients. (A) Principal components analysis revealed distinct immune phenotypes between high‐ and low‐risk ccRCC patients. (B,C) Gene set enrichment analysis enriched five immune‐related biological processes in the high‐risk ccRCC patients. ccRCC, clear cell renal cell carcinoma

Considering the different prognoses between the high‐risk and low‐risk groups, we investigated whether the local immune phenotype exhibited any difference. Based on the eight most valuable prognostic IRGs of the prediction model, principal components analysis (PCA) was conducted using the “pca3d” package. High‐ and low‐risk ccRCC patients were clearly distributed into two subgroups (Figure 7A). GSEA was performed with the high‐ and low‐risk ccRCC patients belonging to the total TCGA cohort. We used c2.cp.kegg.v7.0.symbols.gmt file as the reference gene set and *p* < 0.05 as the threshold criterion. Results revealed that the high‐risk ccRCC patients were enriched with five immune‐related biological pathways: primary immunodeficiency (NES = 1.907, size = 35), intestinal immune network for immunoglobulin A (IgA) production (NES = 1.792, size = 43), cytokine–cytokine receptor interaction (NES = 1.615, size = 236), complement and coagulation cascades (NES = 1.406, size = 69), and natural killer cell‐mediated cytotoxicity (NES = 1.413, size = 117; Figure 7B,C; Table S6). In contrast, no immune‐related biological pathway was related with the low‐risk ccRCC patients (Table S7). These results suggest that an intense local immune phenotype was conferred in high‐risk patients.

### 3.8 Analysis of immune microenvironment and immune checkpoints

Immune and stromal scores for the total TCGA cohort were calculated using the ESTIMATE algorithm. Patients were classified into two subgroups (high‐ and low‐score groups) based on the median value. Survival analysis based on the immune score showed that patients in the high‐score group had poorer prognosis than those in the low‐score group. However, survival analysis based on the stromal score showed no significant differences (Figure S6A). The immune prediction model calculated risk scores of ccRCC patients were positively correlated with the immune and stromal scores (Figure S6B). Moreover, we found that the ccRCC patients in the high‐risk group had significantly higher immune and stromal scores than those in the low‐risk group (Figure S6C).

**FIGURE 8:**
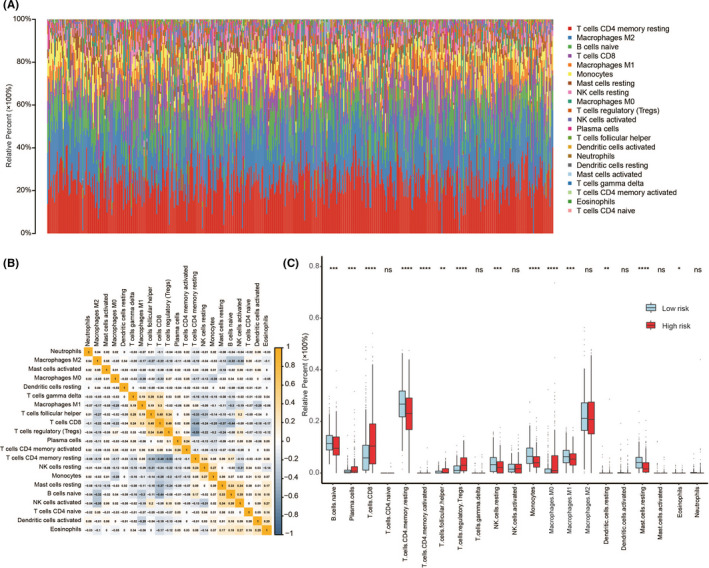
Summary of inferred immune cell subsets. (A) The relative proportions of 21 immune cell types in each sample of ccRCC. (B) Correlation matrix of relative proportions of the 21 immune cell types. (C) The difference of immune cell infiltration in each sample between high‐risk and low‐risk patients. ^*^ *p* < 0.05, ^**^ *p* < 0.01, ^***^ *p* < 0.001, ^****^ *p* < 0.0001. ccRCC, clear cell renal cell carcinoma; ns, no statistical difference

CIBERSORT method used to evaluate the relative proportions of 22 immune cells in ccRCC patients (Figure 8A) detected 21 immune cell types; memory B cell did not appear in any ccRCC patient. Proportions of the 21 immune cell types varied in each patient, suggesting that the infiltration variations of immune cells might be an intrinsic characteristic phenotype of each ccRCC patient. We also evaluated the correlation among the 21 immune cell types; results are shown in Figure 8B. The proportions of 21 immune cell types showed a weak to moderate correlation. The proportions of these immune cells were compared between the high‐ and low‐risk ccRCC patients (*p* < 0.05, Figure 8C). Immune cells, including regulatory T cells (Tregs), follicular helper T cells, M0 macrophages, plasma cells, and CD8^+^ T cells were higher in high‐risk ccRCC patients, while proportions of immune cells, including naive B cells, resting CD4 memory T cells, resting natural killer (NK) cells, monocytes, M1 macrophages, and resting mast cells were lower, suggesting an immunosuppressive microenvironment. However, the activated NK cells showed no significant differences. Different immune landscape between the high‐ and low‐risk groups suggested that the immune‐related prediction model can be used as an indicator of immune status.

**FIGURE 9:**
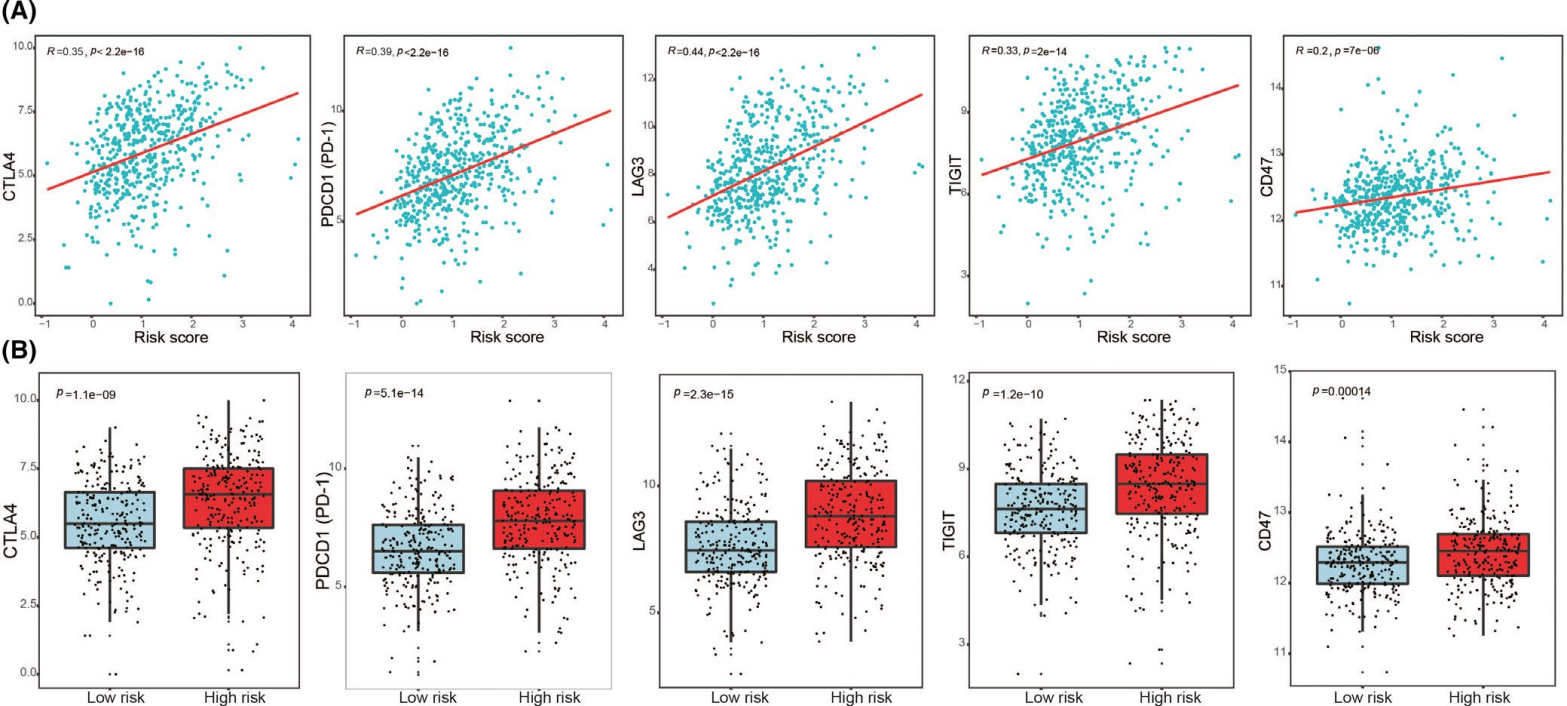
Association with critical immune checkpoints. (A) Risk scores of ccRCC patients were positively correlated with the expression levels of critical immune checkpoint genes (*CTLA‐4*, *PD‐1*, *LAG‐3*, *TIGIT*, and *CD47*). (B) High‐risk ccRCC patients had significantly higher expression levels of *CTLA‐4*, *PD‐1*, *LAG‐3*, *TIGIT*, and *CD47* compared to the low‐risk patients. ccRCC, clear cell renal cell carcinoma

The expression of immune checkpoints and biomarkers for immunotherapy play an important role in the establishment of tumor immunosuppressive microenvironment.^7^ A positive relationship was observed between risk scores of ccRCC patients and immune checkpoint proteins (cytotoxic T‐lymphocyte‐associated protein 4 [*CTLA‐4*], programmed cell death protein 1 [*PD‐1*], lymphocyte‐activation gene 3 [*LAG‐3*], T‐cell immunoreceptor with immunoglobulin and immunoreceptor tyrosine‐based inhibition motif domains [*TIGIT*], and cluster of differentiation 47 [*CD47*]) (*p* < 0.05; Figure 9A). Moreover, the expression levels of immune checkpoints were compared for the patients of high‐ and low‐risk groups; higher expression was observed in high‐risk patients (*p* < 0.05; Figure 9B), suggesting prevalence of an immunosuppressive microenvironment.

### 3.9 Development of a nomogram based on the immune‐related prediction model and clinical factors

**FIGURE 10:**
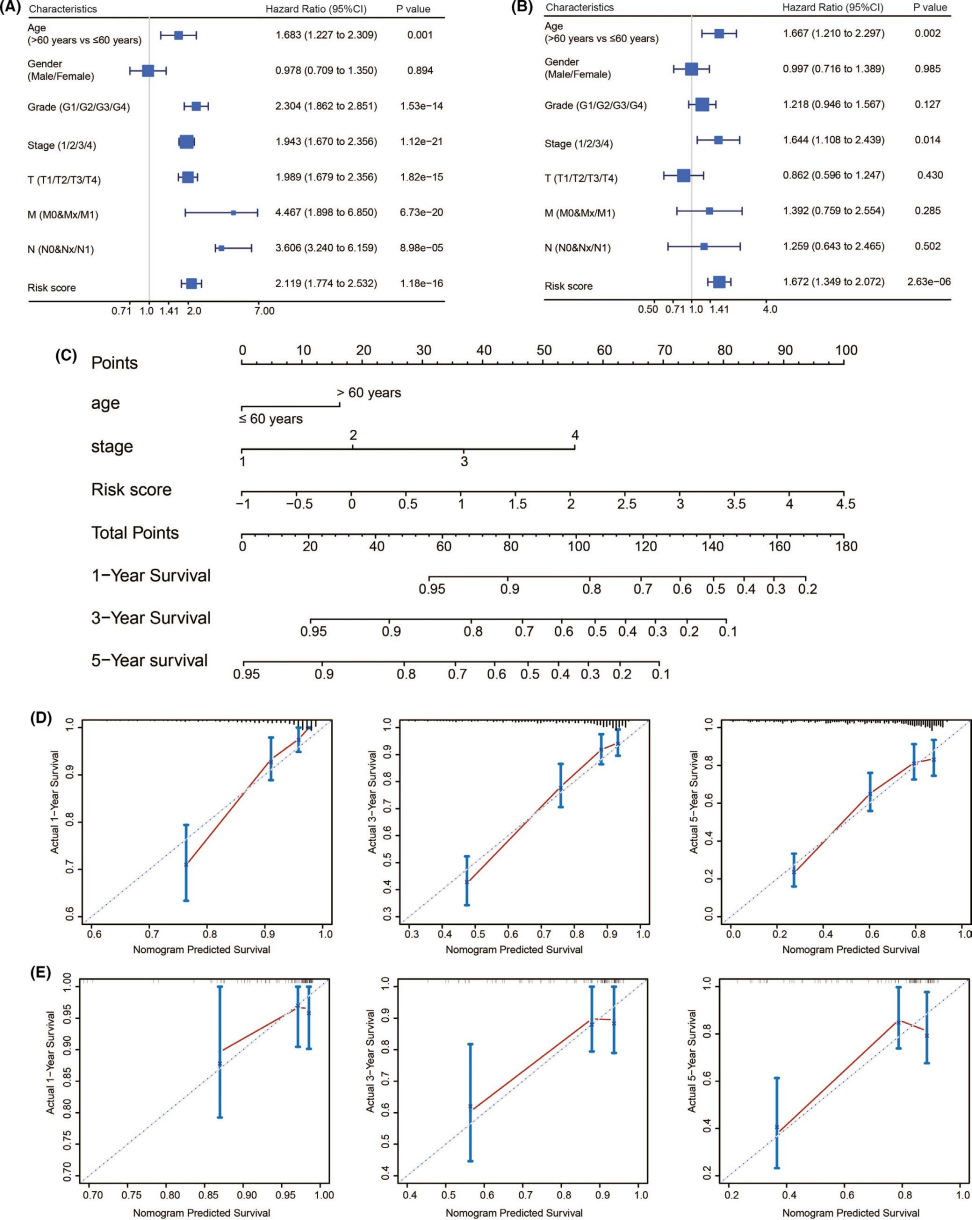
Relationship between the model and other clinical information. Univariate (A) and multivariate (B) Cox regression analysis of the relationship between the immune prediction model and clinicopathological factors. (C) A nomogram combining the immune prediction model with clinical factors for prediction of 1‐, 3‐, and 5‐year survival rate. (D) Calibration plots of the nomogram showed good accuracy of prediction for survival at 1, 3, and 5 years. (E) Calibration plots of the nomogram for survival at 1, 3, and 5 years in the International Cancer Genomics Consortium dataset

To provide a quantitative method for prediction of ccRCC patients’ prognosis, a nomogram integrating clinical factors of ccRCC patients and prediction model was established. Clinical factors that were included are age, sex, Fuhrman grade, pathological stages, pathological T stage, pathological N stage, and pathological M stage. Univariate Cox analysis showed that the prediction model and clinical factors, including age, Fuhrman grade, pathological stages, pathological T stage, pathological N stage, and pathological M stage, were risk factors for overall survival (*p* < 0.05; Figure 10A). Multivariate Cox analysis showed that the prediction model and clinical factors including, age, and pathological stages were independent prognostic factors (*p* < 0.05; Figure 10B). The *C*‐indexes were calculated to evaluate the predictive power of these factors. The combination of risk score based on the prediction model and clinical factors had higher *C*‐index values (0.772; 95% confidence interval: 0.737–0.807) than risk score or clinical factors alone (Table 2), indicating that the incorporated factors influencing risk score of the model and clinical factors could improve the predictive accuracy of overall survival in ccRCC patients. A nomogram was then constructed by integrating the independent clinical parameters (age and pathological stages) and prediction model (Figure 10C). The calibration curves indicated good performance between the predicted and the observed values for 1‐, 3‐, and 5‐year survival (Figure 10D). In addition, the calibration curves for the nomogram validated in the ICGC dataset also showed similar results (Figure 10E).

**TABLE 2:** Comparison of the predictive powers of the prediction models in the total cohorts (*n* = 520)

Factors	Overall survival	
	*C*‐index	95% CI
Age	0.570	0.529–0.611
Stage	0.735	0.696–0.774
Risk score	0.668	0.623–0.713
Age + stage	0.755	0.718–0.792
Risk score + age + stage	0.772	0.737–0.807

Abbreviations: CI, confidence interval; *C*‐index, Harrell's concordance index.

## 4 DISCUSSION

The relationship between gene mutation and immune microenvironment is important for understanding the mechanism and evaluating the effectiveness of ccRCC immunotherapy. Previous large‐scale sequencing data identified *PBRM1* mutation as the second most frequent mutation event in ccRCC, occurring in up to 41% of ccRCC tumors^8^; these findings were confirmed in this study. Previous studies suggested that *PBRM1* mutation enhances the sensitivity of tumor cells to immunotherapeutic drugs and hence can be used as a new biomarker to evaluate the effectiveness of immunotherapy.^12^, ^13^ Tumor cells with *PBRM1* mutation produce a large number of chemokines that recruit effector T cells into tumors.^13^ However, the key *PBRM1* target genes involved in cytokine production and inflammation pathways are yet to be identified. The mechanism of effect of *PBRM1* mutation on the immune microenvironment of ccRCC is also unknown. Therefore, in this study the immune‐related biomarkers associated with *PBRM1* status were screened and their relationship with patient prognosis was explored. Moreover, an immune‐related prediction model for ccRCC patients using immunological biomarkers associated with *PBRM1* mutation was constructed and validated to further evaluate the association between the model and immune phenotype.

In the current study, GSEA showed different immune status of *PBRM1* ^WT^ and *PBRM1* ^MUT^ ccRCC patients. Differential expressions of the inflammatory and immune molecules between the two subgroups suggest that *PBRM1* mutation might be associated with inflammatory and immune responses. We systematically analyzed the association between *PBRM1* mutation status and clinicopathological factors and showed that patients with *PBRM1* mutation tended to have a relatively higher proportion of advanced stage and high‐grade tumors. Then, eight most valuable candidate biomarkers associated with *PBRM1* mutation (*NPR3*, *MDK*, *IFNE*, *NTF4*, *PTGER1*, *GAL*, *FGF23*, and *CXCL13*) were identified. Notably, all these biomarkers with prognostic value were cytokines or cytokine receptors, playing important roles in chemotaxis, angiogenesis, and mediating inflammation and immune cell trafficking.^28^ Of these genes, *CXCL13* promoted progression and predicted poor prognosis in ccRCC patients.^29^ However, to unravel the role of *CXCL13* in the immune microenvironment of ccRCC, further investigations are warranted. *NPR3* plays a pivotal role in the proliferation and metastasis of ccRCC by negatively regulating the p38‐MAPK signaling pathway.^30^ MDK, a heparin‐binding growth factor, is strongly expressed in the majority of human malignant tumors.^31^ Moreover, it is reported that downregulation of *MDK* induces cisplatin resistance in RCC.^32^ Other genes were identified in this study for the first time to have prognostic value in ccRCC patients. *IFNE* deletion is common in bladder cancer cells.^33^ *NTF4*, significantly overexpressed in colorectal cancer, was associated with poor overall survival and advanced TNM stage. *NTF4* promoted tumorigenesis and development of colorectal cancer through autophagy regulation.^34^ PTGER1 upregulates the aldosterone‐producing adenoma; its expression remains associated with DNA methylation.^35^ Recent studies identified that overexpression of *GAL* in colon cancer and silencing of *GAL* could result apoptosis and enhanced chemotherapy effects.^36^, ^37^ FGF23 has been recently identified to have implications in the pathogenesis of oncogenic osteomalacia and hence could be used as a potential new clinical marker for the diagnosis and clinical management of oncogenic osteomalacia.^38^ In present study, we showed that these genes were associated with different immune cells in the local immune microenvironment of ccRCC patient. We found high expressions of FGF23 and low expressions of PTGER1 in ccRCC compared with matched normal tissues. These genes having great potential as individual targets improved the treatment strategies in the era of ongoing immunotherapeutic revolution. In addition, gene functional enrichment analysis, based on differentially expressed IRGs and survive‐associated IRGs, showed “cytokine–cytokine receptor interaction” as the significantly enriched biological process. Previous studies reported that an enhanced inflammatory microenvironment remains frequently associated with tumorigenesis and actively participates in the pathogenesis of ccRCC.^39^, ^40^, ^41^ In this study, we hypothesized that “cytokine–cytokine receptor interaction” plays a crucial role in the pathogenesis of ccRCC, confirming that cytokines and inflammatory processes are essential for the initiation and progression of ccRCC.^28^, ^42^

An effective prediction model to monitor the immune status and predict clinical outcomes of ccRCC patients was developed and validated using the eight candidate biomarkers. Survival analysis showed distinctly different prognoses between the high‐ and low‐risk ccRCC patients. At the same time, the prediction model showed good diagnostic sensitivity and accuracy. The prediction model also applies to specific ccRCC patients with different *PBRM1* status, *PBRM1* ^MUT^ and *PBRM1* ^WT^ TCGA cohorts, demonstrating favorable clinical predictive ability. In addition, the predictive performance of the prediction model for ccRCC patients was independent of the *PBRM1* status, suggesting wide application of this prediction model. We also found that patients with higher risk score in our prediction model tended to have advanced stage and high‐grade tumors with node metastasis, distant metastasis, and poor prognosis. These results indicated that the prediction model in our study has a clinical application potential comparable to the traditional clinical factors. We integrated the prediction model and clinical characteristics to establish a novel nomogram model. The nomogram took advantage of the complementary values of clinical characteristics and prediction model and provided a superior estimation of overall survival. However, the biological functions and potential molecular mechanisms of these eight genes in the immune response remain unknown; future studies are warranted to explore the associations between expression of these eight genes and *PBRM1* mutation status using real‐world samples. Unraveling of the potential molecular mechanisms may accelerate their clinical application in ccRCC.

Recent studies have identified the vital role of local immune cell infiltrates in ccRCC tissues and different immune cell compositions have been linked to the prognosis of ccRCC patients.^43^ Several studies have sought to depict the immune infiltration landscape in ccRCC to identify novel immune characterization with prognostic potential.^44^, ^45^, ^46^ In the present study, the effect of *PBRM1* mutation status was considered as a potential biomarker in ccRCC. We selected *PBRM1* target genes and IRGs. Based on the immune‐associated prediction model, PCA showed a distinct subgroup and GSEA showed an enhanced local immune phenotype in high‐risk ccRCC patients, including enrichment of “natural killer cell mediated cytotoxicity” and “intestinal immune network for IgA production” pathways. The enriched “intestinal immune network for IgA production” pathway was associated with increased B cells or plasma cells, which was confirmed by the infiltrating immune cells analysis. We found significantly increased plasma cells in high‐risk patients. The immune microenvironment of ccRCC was estimated using the ESTIMATE algorithm. We focused on the difference in immune and stromal scores correlated with ccRCC subtypes and indicated that distinctly different immune phenotypes remain associated with ccRCC subtypes. We further explored the difference in immune cell infiltration between the ccRCC subtypes to reflect the status of the immune microenvironment in the ccRCC patients. In the current study, the infiltration levels of immune cells varied from patient to patient, indicating that the immune microenvironment is an inherent feature of each patient. The characteristics of tumor‐infiltrating immune cells between high‐ and low‐risk patients indicated that patients in the former group had significantly higher proportions of immunosuppressive cells (Tregs, M0 macrophages, and follicular helper T cells) and lower fractions of immunocompetent cells (naive B cells, resting CD4 memory T cells, resting NK cells, monocytes, M1 macrophages, and resting mast cells), suggesting the prevalence of an immunosuppressive tumor microenvironment. Higher CD8^+^ T‐cell infiltration, a crucial immune cell in antitumor immune effects,^47^ was also found in the high‐risk patients. The observation can be explained by the fact that antitumor immune effects are counterbalanced by a strong immunosuppressive environment in high‐risk patients.^44^, ^48^, ^49^ In addition, immune checkpoints (*CTLA‐4*, *PD‐1*, *LAG‐3*, *TIGIT*, and *CD47*) were evaluated between the high‐ and low‐risk patients; higher expression was observed in high‐risk patients, suggesting prevalence of a stronger immunosuppressive environment. These findings suggest that high‐risk patients with enhanced expression of immune checkpoints might benefit more from immune checkpoint inhibitors than low‐risk patients. The strong immunosuppressive environment in high‐risk patients might be the possible reason for the poor prognosis. Therefore, our prediction model was not only efficient in determining the immune status of patients, but it also serves as a useful tool to stratify patients, thus increasing the effectiveness of immunotherapy and resulting in a better prognosis.

Based on the *PBRM1* mutation status and immune genes, our research provided new insights into the ccRCC immune microenvironment and immune‐related therapies. Compared with previous studies, our prediction model had a number of strengths. First, our prediction model was sufficiently validated in an internal and two external datasets. Second, our prediction model associated with *PBRM1* mutation had a significant biological background, while *PBRM1* mutation was associated with the effectiveness of immunotherapy. This is the essential difference between our prediction model and previous prognostic models. However, the present study had some limitations. This study is a retrospective study. Prospective clinical trials are needed to validate the prediction performance of the model and to evaluate its clinical applicability for better management of ccRCC. The expressions, biological functions, prognostic values, and associations with tumor immune microenvironment of these genes are required to be confirmed individually and in combination in the actual clinical samples. Moreover, the present study was constructed based on mRNA sequencing, multi‐omics sequencing data associated with *PBRM1* mutation might provide more comprehensive and objective results. However, the study was limited owing to its retrospective nature. Prospective clinical trials are needed to validate the prediction performance of the model and to evaluate its clinical applicability for better management of ccRCC.

In summary, an eight gene immune prediction model incorporating *PBRM1* status was constructed and validated in this study. To the best of our knowledge, this is the first prediction model associated with *PBRM1* mutation status and immunity. The prediction model could classify the ccRCC patients into subgroups with distinct prognoses. It can be a promising tool to determine the immune status and stratify patients, thus guiding immunotherapy in ccRCC patients. A nomogram, combining the prediction model and the complementary value of clinical factors was constructed, which could be a useful tool to predict prognosis and guide clinical practice.

## CONFLICT OF INTEREST

The authors declared that they have no competing interests.

## Supporting information

TABLE S1

TABLE S2

TABLE S3

TABLE S4

TABLE S5

TABLE S6

TABLE S7

FIGURE S1

FIGURE S2

FIGURE S3

FIGURE S4

FIGURE S5

FIGURE S6

## Data Availability

The data for the current study are available from the corresponding author upon reasonable request.

## References

[1] Siegel RL , Miller KD , Jemal A . Cancer statistics, 2019. CA Cancer J Clin. 2019;69(1):7‐34. 30620402 10.3322/caac.21551

[2] Leibovich BC , Lohse CM , Crispen PL , et al. Histological subtype is an independent predictor of outcome for patients with renal cell carcinoma. J Urol. 2010;183(4):1309‐1315. 20171681 10.1016/j.juro.2009.12.035

[3] Weinstock M , McDermott D . Targeting PD‐1/PD‐L1 in the treatment of metastatic renal cell carcinoma. Ther Adv Urol. 2015;7(6):365‐377. 26622321 10.1177/1756287215597647 PMC4647139

[4] Topalian SL , Hodi FS , Brahmer JR , et al. Safety, activity, and immune correlates of anti‐PD‐1 antibody in cancer. N Engl J Med. 2012;366(26):2443‐2454. 22658127 10.1056/NEJMoa1200690 PMC3544539

[5] Brahmer JR , Tykodi SS , Chow LQM , et al. Safety and activity of anti‐PD‐L1 antibody in patients with advanced cancer. N Engl J Med. 2012;366(26):2455‐2465. 22658128 10.1056/NEJMoa1200694 PMC3563263

[6] Geissler K , Fornara P , Lautenschläger C , et al. Immune signature of tumor infiltrating immune cells in renal cancer. Oncoimmunology. 2015;4(1):e985082. 25949868 10.4161/2162402X.2014.985082 PMC4368143

[7] Kawashima A , Kanazawa T , Goto K , et al. Immunological classification of renal cell carcinoma patients based on phenotypic analysis of immune check‐point molecules. Cancer Immunol Immunother. 2018;67(1):113‐125. 28975380 10.1007/s00262-017-2060-5 PMC11028191

[8] Varela I , Tarpey P , Raine K , et al. Exome sequencing identifies frequent mutation of the SWI/SNF complex gene PBRM1 in renal carcinoma. Nature. 2011;469(7331):539‐542. 21248752 10.1038/nature09639 PMC3030920

[9] Gerlinger M , Horswell S , Larkin J , et al. Genomic architecture and evolution of clear cell renal cell carcinomas defined by multiregion sequencing. Nat Genet. 2014;46(3):225‐233. 24487277 10.1038/ng.2891 PMC4636053

[10] Hargreaves DC , Crabtree GR . ATP‐dependent chromatin remodeling: genetics, genomics and mechanisms. Cell Res. 2011;21(3):396‐420. 21358755 10.1038/cr.2011.32 PMC3110148

[11] Reisman D , Glaros S , Thompson EA . The SWI/SNF complex and cancer. Oncogene. 2009;28(14):1653‐1668. 19234488 10.1038/onc.2009.4

[12] Miao D , Margolis CA , Gao W , et al. Genomic correlates of response to immune checkpoint therapies in clear cell renal cell carcinoma. Science. 2018;359(6377):801‐805. 29301960 10.1126/science.aan5951 PMC6035749

[13] Pan D , Kobayashi A , Jiang P , et al. A major chromatin regulator determines resistance of tumor cells to T cell‐mediated killing. Science. 2018;359(6377):770‐775. 29301958 10.1126/science.aao1710 PMC5953516

[14] Bhattacharya S , Andorf S , Gomes L , et al. ImmPort: disseminating data to the public for the future of immunology. Immunol Res. 2014;58(2‐3):234‐239. 24791905 10.1007/s12026-014-8516-1

[15] Chen B , Khodadoust MS , Liu CL , et al. Profiling tumor infiltrating immune cells with CIBERSORT. Methods Mol Biol. 2018;1711:243‐259. 29344893 10.1007/978-1-4939-7493-1_12 PMC5895181

[16] Law CW , Chen Y , Shi W , et al. voom: precision weights unlock linear model analysis tools for RNA‐seq read counts. Genome Biol. 2014;15(2):R29. 24485249 10.1186/gb-2014-15-2-r29 PMC4053721

[17] Robinson MD , McCarthy DJ , Smyth GK . edgeR: a bioconductor package for differential expression analysis of digital gene expression data. Bioinformatics. 2010;26(1):139‐140. 19910308 10.1093/bioinformatics/btp616 PMC2796818

[18] Tibshirani R . The lasso method for variable selection in the Cox model. Stat Med. 1997;16(4):385‐395. 9044528 10.1002/(sici)1097-0258(19970228)16:4<385::aid-sim380>3.0.co;2-3

[19] Lossos IS , Czerwinski DK , Alizadeh AA , et al. Prediction of survival in diffuse large‐B‐cell lymphoma based on the expression of six genes. N Engl J Med. 2004;350(18):1828‐1837. 15115829 10.1056/NEJMoa032520

[20] Chen H‐Y , Yu S‐L , Chen C‐H , et al. A five‐gene signature and clinical outcome in non‐small‐cell lung cancer. N Engl J Med. 2007;356(1):11‐20. 17202451 10.1056/NEJMoa060096

[21] Heagerty PJ , Lumley T , Pepe MS . Time‐dependent ROC curves for censored survival data and a diagnostic marker. Biometrics. 2000;56(2):337‐344. 10877287 10.1111/j.0006-341x.2000.00337.x

[22] Huang DW , Sherman BT , Lempicki RA . Systematic and integrative analysis of large gene lists using DAVID bioinformatics resources. Nat Protoc. 2009;4(1):44‐57. 19131956 10.1038/nprot.2008.211

[23] Yoshihara K , Shahmoradgoli M , Martínez E , et al. Inferring tumour purity and stromal and immune cell admixture from expression data. Nat Commun. 2013;4:2612. 24113773 10.1038/ncomms3612 PMC3826632

[24] Newman AM , Liu CL , Green MR , et al. Robust enumeration of cell subsets from tissue expression profiles. Nat Methods. 2015;12(5):453‐457. 25822800 10.1038/nmeth.3337 PMC4739640

[25] Balachandran VP , Gonen M , Smith JJ , et al. Nomograms in oncology: more than meets the eye. Lancet Oncol. 2015;16(4):E173‐E180. 25846097 10.1016/S1470-2045(14)71116-7 PMC4465353

[26] Peña‐Llopis S , Vega‐Rubín‐de‐Celis S , Liao A , et al. BAP1 loss defines a new class of renal cell carcinoma. Nat Genet. 2012;44(7):751‐759. 22683710 10.1038/ng.2323 PMC3788680

[27] Li T , Fan J , Wang B , et al. TIMER: a web server for comprehensive analysis of tumor‐infiltrating immune cells. Cancer Res. 2017;77(21):e108‐e110. 29092952 10.1158/0008-5472.CAN-17-0307 PMC6042652

[28] Lin WW , Karin M . A cytokine‐mediated link between innate immunity, inflammation, and cancer. J Clin Invest. 2007;117(5):1175‐1183. 17476347 10.1172/JCI31537 PMC1857251

[29] Zheng Z , Cai Y , Chen H , et al. CXCL13/CXCR5 axis predicts poor prognosis and promotes progression through PI3K/AKT/mTOR pathway in clear cell renal cell carcinoma. Front Oncol. 2019;8:682. 30723697 10.3389/fonc.2018.00682 PMC6349755

[30] Li J‐K , Chen C , Liu J‐Y , et al. Long noncoding RNA MRCCAT1 promotes metastasis of clear cell renal cell carcinoma via inhibiting NPR3 and activating p38‐MAPK signaling. Mol Cancer. 2017;16(1):111. 28659173 10.1186/s12943-017-0681-0 PMC5490088

[31] Filippou PS , Karagiannis GS , Constantinidou A . Midkine (MDK) growth factor: a key player in cancer progression and a promising therapeutic target. Oncogene. 2020;39(10):2040‐2054. 31801970 10.1038/s41388-019-1124-8

[32] Kawai H , Sato W , Yuzawa Y , et al. Lack of the growth factor midkine enhances survival against cisplatin‐induced renal damage. Am J Pathol. 2004;165(5):1603‐1612. 15509530 10.1016/S0002-9440(10)63417-7 PMC1618674

[33] Nickerson ML , Witte N , Im KM , et al. Molecular analysis of urothelial cancer cell lines for modeling tumor biology and drug response. Oncogene. 2017;36(1):35‐46. 27270441 10.1038/onc.2016.172 PMC5140783

[34] Yang Z , Chen Y , Wei X , et al. Upregulated NTF4 in colorectal cancer promotes tumor development via regulating autophagy. Int J Oncol. 2020;56(6):1442‐1454. 32236587 10.3892/ijo.2020.5027 PMC7170041

[35] Itcho K , Oki K , Kobuke K , et al. Aberrant G protein‐receptor expression is associated with DNA methylation in aldosterone‐producing adenoma. Mol Cell Endocrinol. 2018;461:100‐104. 28870781 10.1016/j.mce.2017.08.019

[36] Stevenson L , Allen WL , Turkington R , et al. Identification of galanin and its receptor GalR1 as novel determinants of resistance to chemotherapy and potential biomarkers in colorectal cancer. Clin Cancer Res. 2012;18(19):5412‐5426. 22859720 10.1158/1078-0432.CCR-12-1780 PMC3463501

[37] Kim KY , Kee MK , Chong SA , et al. Galanin is up‐regulated in colon adenocarcinoma. Cancer Epidemiol Biomarkers Prev. 2007;16(11):2373‐2378. 18006926 10.1158/1055-9965.EPI-06-0740

[38] Nelson AE , Bligh RC , Mirams M , et al. Clinical case seminar: fibroblast growth factor 23: a new clinical marker for oncogenic osteomalacia. J Clin Endocrinol Metab. 2003;88(9):4088‐4094. 12970268 10.1210/jc.2002-021919

[39] Romero JM , Aptsiauri N , Vazquez F , et al. Analysis of the expression of HLA class I, proinflammatory cytokines and chemokines in primary tumors from patients with localized and metastatic renal cell carcinoma. Tissue Antigens. 2006;68(4):303‐310. 17026465 10.1111/j.1399-0039.2006.00673.x

[40] Cabillic F , Bouet‐Toussaint F , Toutirais O , et al. Interleukin‐6 and vascular endothelial growth factor release by renal cell carcinoma cells impedes lymphocyte‐dendritic cell cross‐talk. Clin Exp Immunol. 2006;146(3):518‐523. 17100773 10.1111/j.1365-2249.2006.03212.x PMC1810419

[41] Alberti L , Thomachot MC , Bachelot T , et al. IL‐6 as an intracrine growth factor for renal carcinoma cell lines. Int J Cancer. 2004;111(5):653‐661. 15252833 10.1002/ijc.20287

[42] Lippitz BE . Cytokine patterns in patients with cancer: a systematic review. Lancet Oncol. 2013;14(6):E218‐E228. 23639322 10.1016/S1470-2045(12)70582-X

[43] Chevrier S , Levine JH , Zanotelli VRT , et al. An immune atlas of clear cell renal cell carcinoma. Cell. 2017;169(4):736‐749.e18. 28475899 10.1016/j.cell.2017.04.016 PMC5422211

[44] Hua X , Chen J , Su Y , et al. Identification of an immune‐related risk signature for predicting prognosis in clear cell renal cell carcinoma. Aging (Albany NY). 2020;12(3):2302‐2332. 32028264 10.18632/aging.102746 PMC7041771

[45] Geissler K , Fornara P , Lautenschläger C , et al. Immune signature of tumor infiltrating immune cells in renal cancer. Oncoimmunology. 2015;4(1):e985082. 25949868 10.4161/2162402X.2014.985082 PMC4368143

[46] Giraldo NA , Becht E , Vano Y , et al. Tumor‐infiltrating and peripheral blood t‐cell immunophenotypes predict early relapse in localized clear cell renal cell carcinoma. Clin Cancer Res. 2017;23(15):4416‐4428. 28213366 10.1158/1078-0432.CCR-16-2848

[47] Tykodi SS , Satoh S , Deming JD , et al. CD8(+) T‐cell clones specific for the 5T4 antigen target renal cell carcinoma tumor‐initiating cells in a murine xenograft model. J Immunother. 2012;35(7):523‐533. 22892449 10.1097/CJI.0b013e318261d630 PMC3423552

[48] Matsushita H , Sato Y , Karasaki T , et al. Neoantigen load, antigen presentation machinery, and immune signatures determine prognosis in clear cell renal cell carcinoma. Cancer Immunol Res. 2016;4(5):463‐471. 26980598 10.1158/2326-6066.CIR-15-0225

[49] Chen Y‐P , Zhang YU , Lv J‐W , et al. Genomic analysis of tumor microenvironment immune types across 14 solid cancer types: immunotherapeutic implications. Theranostics. 2017;7(14):3585‐3594. 28912897 10.7150/thno.21471 PMC5596445

