# Supplementary material for: Development and validation of a PBRM1‐associated immune prognostic model for clear cell renal cell carcinoma
Source: Cancer Med. 2021 Sep 18;10(19):6590–609. doi: 10.1002/cam4.4115 (PMC8495284; doi:10.1002/cam4.4115)
Supplement: Supplementary file 4 — TABLE S4 [file CAM4-10-6590-s013.docx]

Table S4. General characteristics of 87 PBRM1-asscociated differently expressed immune-related genes of ccRCC.

| Gene symbol | logFC | FDR | HR | Z-value | P-value |
| --- | --- | --- | --- | --- | --- |
| NPR3 | -1.195265531 | 7.27E-12 | 0.77347544 | -5.693483913 | 1.24E-08 |
| MDK | 1.016846907 | 1.17E-08 | 1.419584329 | 4.716966737 | 2.39E-06 |
| IFNE | 2.392376631 | 8.98E-14 | 1.287358058 | 4.627033701 | 3.71E-06 |
| NTF4 | 2.138467964 | 6.78E-10 | 1.378209593 | 4.589880251 | 4.44E-06 |
| PTGER1 | 1.749487612 | 1.73E-07 | 1.225904842 | 3.935918119 | 8.29E-05 |
| GAL | 2.535066265 | 6.34E-12 | 1.252613615 | 3.880387604 | 0.00010429 |
| FGF23 | 1.022588577 | 0.00926311 | 1.342563096 | 3.733600574 | 0.000188762 |
| CXCL13 | 1.028104741 | 0.000147855 | 1.182534695 | 3.517899375 | 0.000434977 |
| ACVR1C | 1.552250057 | 1.07E-09 | 1.134284558 | 1.930598232 | 0.053532757 |
| ADRB1 | 2.102931178 | 4.60E-18 | 1.044983269 | 0.596720978 | 0.550693684 |
| ANGPTL1 | 1.95174879 | 5.87E-18 | 0.968801582 | -0.509768452 | 0.61021369 |
| ANGPTL3 | 1.706572828 | 1.75E-09 | 0.901074804 | -2.252301997 | 0.024303194 |
| ANGPTL7 | 2.607698442 | 9.66E-16 | 1.036236486 | 0.620986657 | 0.534608402 |
| ARTN | 1.106023171 | 1.09E-08 | 1.359156358 | 4.710400195 | 2.47E-06 |
| AVP | 1.238704019 | 0.000370701 | 1.202834179 | 2.343458936 | 0.019105865 |
| BMP3 | -1.956211937 | 8.33E-19 | 1.034582875 | 0.469962437 | 0.638381855 |
| BMP5 | -1.013347464 | 0.001174444 | 0.931216637 | -1.719499608 | 0.085523439 |
| BMP7 | 4.216369734 | 3.93E-27 | 1.065086148 | 1.41631437 | 0.156683482 |
| BMPR1B | 1.241539545 | 0.000174104 | 1.114465779 | 2.429623149 | 0.015114529 |
| C5 | 1.181927408 | 2.33E-13 | 1.527189111 | 3.04378279 | 0.002336238 |
| CALCA | 2.974336695 | 7.16E-07 | 0.978215064 | -0.295636244 | 0.767507892 |
| CBLC | 1.445934987 | 8.17E-11 | 1.08061568 | 1.239286248 | 0.21523951 |
| CCK | 1.884180556 | 1.71E-06 | 0.795129807 | -1.532758049 | 0.12533548 |
| CCL11 | 1.045533737 | 0.000477531 | 1.17813401 | 3.229116767 | 0.001241732 |
| CCL19 | 1.282888952 | 7.99E-06 | 1.099260135 | 2.150666847 | 0.031502505 |
| CCL21 | 1.894956495 | 7.91E-09 | 1.054681505 | 1.463921985 | 0.143215266 |
| CGA | 3.344409162 | 1.04E-07 | 1.165257661 | 2.424592982 | 0.015325558 |
| CHGB | 1.044261385 | 7.49E-05 | 1.062046692 | 1.058285675 | 0.289925222 |
| CR2 | 2.520663121 | 3.88E-15 | 1.078445917 | 1.463750702 | 0.143262077 |
| CRH | 3.927902379 | 1.21E-12 | 1.176733647 | 1.827131005 | 0.067680075 |
| CRHR1 | 2.608295861 | 9.97E-10 | 1.054184008 | 1.232775063 | 0.217659698 |
| CSPG5 | 1.052506294 | 7.15E-12 | 1.201329622 | 1.966731418 | 0.049214183 |
| CXCL5 | -1.478627679 | 1.41E-06 | 1.121587332 | 3.501325017 | 0.000462951 |
| CYSLTR2 | 1.296116606 | 6.10E-06 | 0.955350816 | -0.805171084 | 0.420720961 |
| DEFB1 | 2.030907527 | 8.48E-15 | 0.969193488 | -0.574782761 | 0.565438227 |
| ESRRB | 1.038718354 | 3.85E-06 | 1.047384854 | 0.522348645 | 0.601427605 |
| FAM3B | 2.253388221 | 2.98E-19 | 1.017449633 | 0.287444414 | 0.773772058 |
| FGF10 | 1.33419647 | 0.000136315 | 1.010325726 | 0.147134826 | 0.883025602 |
| FGF16 | 1.050944569 | 0.00132863 | 1.357916092 | 3.04929256 | 0.00229381 |
| FGF19 | 1.028936426 | 0.034715339 | 1.266766155 | 2.222149757 | 0.026273187 |
| FGF7 | 1.506059408 | 4.34E-09 | 1.090483076 | 1.787408111 | 0.073871552 |
| FGF9 | 3.034021358 | 3.79E-13 | 0.995741162 | -0.090337884 | 0.928018716 |
| FSHR | 1.51430899 | 3.70E-08 | 0.943741089 | -0.797181304 | 0.425345742 |
| GALP | 1.014544275 | 0.042037473 | 1.177679936 | 1.710221583 | 0.087224906 |
| GCG | 2.858987177 | 4.99E-07 | 1.343590826 | 2.952256354 | 0.003154609 |
| GCGR | 4.507482834 | 9.29E-17 | 1.096098892 | 2.426501766 | 0.015245176 |
| GDNF | 2.676314748 | 1.50E-19 | 1.009315901 | 0.137617524 | 0.890542706 |
| GREM1 | 1.199961743 | 2.84E-05 | 1.153794724 | 3.152984638 | 0.001616103 |
| GREM2 | -1.4546522 | 2.68E-05 | 1.140237694 | 3.527144686 | 0.000420067 |
| GRP | 2.76745669 | 2.69E-10 | 1.09291234 | 1.6544826 | 0.098029496 |
| HTR3B | 2.714295099 | 1.37E-07 | 0.98494031 | -0.129155498 | 0.897234609 |
| IGF2 | 2.405632408 | 6.11E-12 | 1.012432611 | 0.258291325 | 0.796182081 |
| IL11 | 1.512062827 | 2.04E-07 | 1.301474998 | 4.342591394 | 1.41E-05 |
| IL12B | 1.282390677 | 3.06E-08 | 0.990745268 | -0.134170633 | 0.893267648 |
| IL20 | 1.03009856 | 0.006983296 | 0.997263062 | -0.022952563 | 0.981688112 |
| IL20RA | 2.131271407 | 2.31E-10 | 1.193434828 | 3.625475772 | 0.00028843 |
| INHA | 1.872289704 | 1.71E-07 | 1.132996502 | 3.005244936 | 0.002653671 |
| LEFTY1 | 2.823919416 | 1.63E-18 | 1.065939498 | 1.432842317 | 0.151902905 |
| LEFTY2 | 1.430407438 | 4.99E-08 | 0.995025243 | -0.083166791 | 0.933718919 |
| LHCGR | 5.314277251 | 9.99E-26 | 1.042103592 | 0.863634252 | 0.387788842 |
| MIA | 1.794377851 | 8.66E-10 | 1.185277279 | 2.327736963 | 0.019926075 |
| MTNR1A | 1.047209502 | 0.00042781 | 0.981978146 | -0.24745548 | 0.804555742 |
| NDP | 3.79849334 | 1.43E-30 | 1.066165011 | 1.164978056 | 0.244027875 |
| NPPB | 1.000183361 | 0.011387702 | 1.229523097 | 2.289475818 | 0.022051721 |
| NPPC | 1.387897643 | 2.36E-05 | 0.964487666 | -0.629534816 | 0.528998983 |
| NPY | 1.150533243 | 3.38E-06 | 0.826741921 | -1.754250502 | 0.079387593 |
| NR0B1 | 1.86844812 | 0.001468868 | 1.119730974 | 2.753429742 | 0.005897443 |
| NR0B2 | 4.857091177 | 7.81E-17 | 0.981535568 | -0.315481429 | 0.752396149 |
| NR5A1 | -1.600485203 | 1.08E-06 | 1.038921902 | 0.48823073 | 0.62538642 |
| OGN | 1.410606882 | 4.08E-06 | 0.98344008 | -0.392709453 | 0.694534088 |
| OPRK1 | 1.419293208 | 0.000130893 | 1.109450461 | 0.955209099 | 0.339471965 |
| PAK6 | 1.763280884 | 4.75E-11 | 1.048692625 | 0.580631684 | 0.561488714 |
| PDGFRA | 1.114613214 | 2.51E-06 | 1.129337909 | 2.207123349 | 0.027305443 |
| PPY | 1.079482626 | 0.00057696 | 1.34335739 | 3.272385852 | 0.001066439 |
| PRLR | 1.039441727 | 4.00E-07 | 0.957651887 | -0.599958711 | 0.548533753 |
| PTGDS | 2.521223167 | 1.40E-18 | 1.160825907 | 2.412644886 | 0.015837241 |
| PYY | 1.831140643 | 6.97E-14 | 1.242174036 | 3.469404595 | 0.000521613 |
| RAET1L | 1.169890888 | 1.89E-05 | 1.068447536 | 0.798252795 | 0.424723806 |
| REG1A | 1.826562588 | 1.29E-08 | 0.987799886 | -0.373976072 | 0.708422118 |
| RORB | -2.148437494 | 5.09E-11 | 1.225579759 | 3.519788337 | 0.000431891 |
| SCG2 | -1.524035806 | 4.12E-13 | 1.118752963 | 2.136607481 | 0.032629931 |
| SEMA3E | 2.779795722 | 5.05E-12 | 1.139757458 | 3.545539718 | 0.00039181 |
| SLIT2 | 1.267313074 | 1.13E-11 | 1.041638199 | 0.544171239 | 0.586323641 |
| SSTR5 | 1.12047605 | 0.00046897 | 1.081929827 | 1.352237576 | 0.176299327 |
| TAC1 | 3.850604249 | 2.71E-18 | 0.989406059 | -0.179826254 | 0.857288972 |
| TDGF1 | 1.053528876 | 0.009852218 | 1.017740556 | 0.359718207 | 0.719057875 |
| THPO | 1.906476873 | 7.72E-20 | 1.058238286 | 1.117089907 | 0.263955885 |
