# Supplementary material for: Development and validation of a PBRM1‐associated immune prognostic model for clear cell renal cell carcinoma
Source: Cancer Med. 2021 Sep 18;10(19):6590–609. doi: 10.1002/cam4.4115 (PMC8495284; doi:10.1002/cam4.4115)
Supplement: Supplementary file 5 — TABLE S5 [file CAM4-10-6590-s003.docx]

Table S5: Basic characteristics of eight immune-related genes with prognostic value.

| Gene | Description | Function | Cytoband |
| --- | --- | --- | --- |
| NPR3 | natriuretic peptide receptor 3 | peptide receptor activity | 5p13.3 |
| MDK | midkine | heparin binding | 11p11.2 |
| IFNE | interferon epsilon | cytokine activity | 9p21.3 |
| NTF4 | neurotrophin 4 | growth factor activity | 19q13.33 |
| PTGER1 | PTGER1 | prostaglandin E receptor activity | 19p13.12 |
| GAL | galanin and GMAP prepropeptide | neuropeptide hormone activity | 11q13.2 |
| FGF23 | fibroblast growth factor 23 | growth factor activity | 12p13.32 |
| CXCL13 | C-X-C motif chemokine ligand 13 | chemokine activity | 4q21.1 |
