# Supplementary figures and images for: Development and validation of a PBRM1‐associated immune prognostic model for clear cell renal cell carcinoma
Source: Cancer Med. 2021 Sep 18;10(19):6590–609. doi: 10.1002/cam4.4115 (PMC8495284; doi:10.1002/cam4.4115)

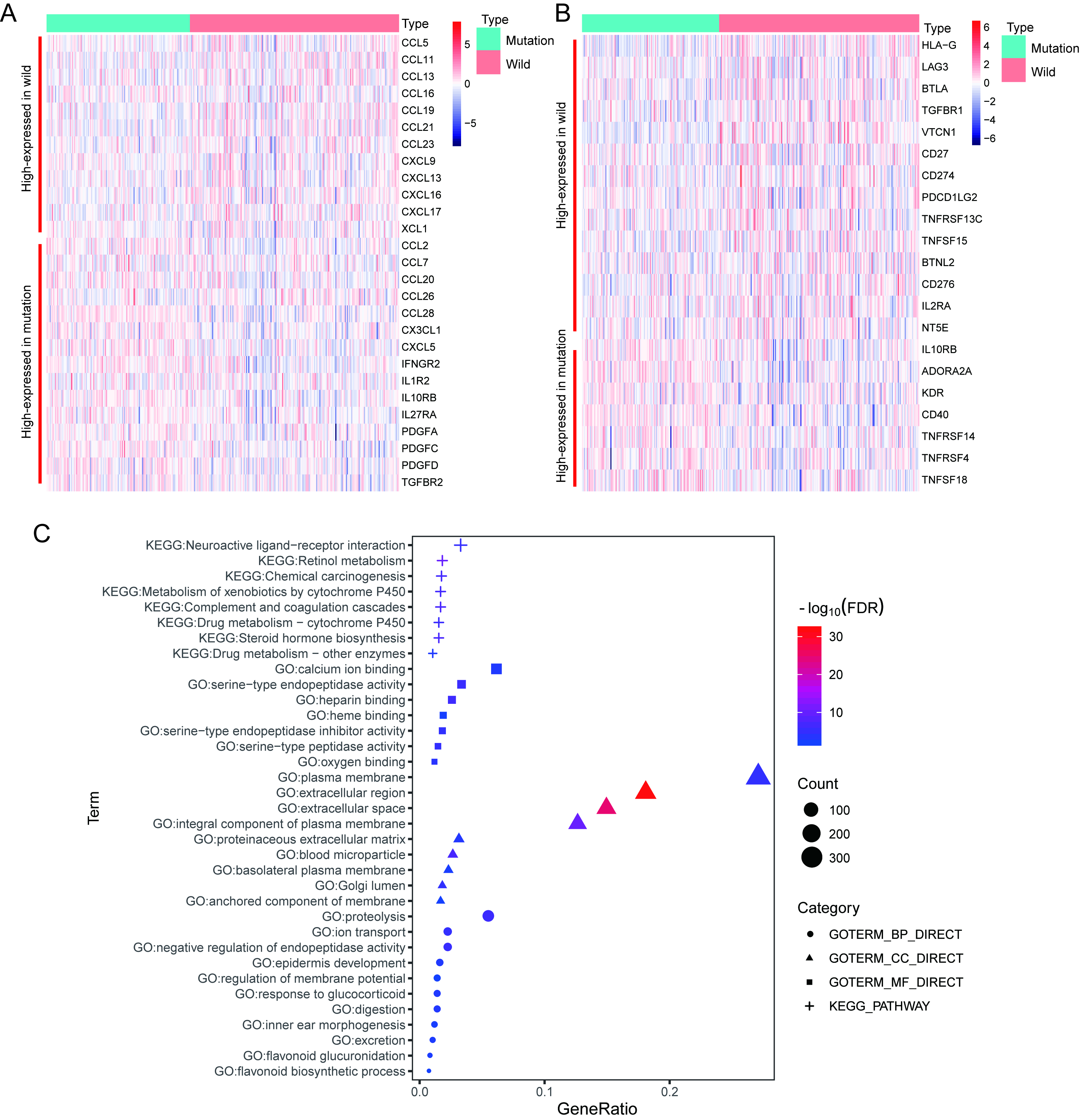

Supplement: Supplementary file 8 — FIGURE S1 [file CAM4-10-6590-s010.tif]

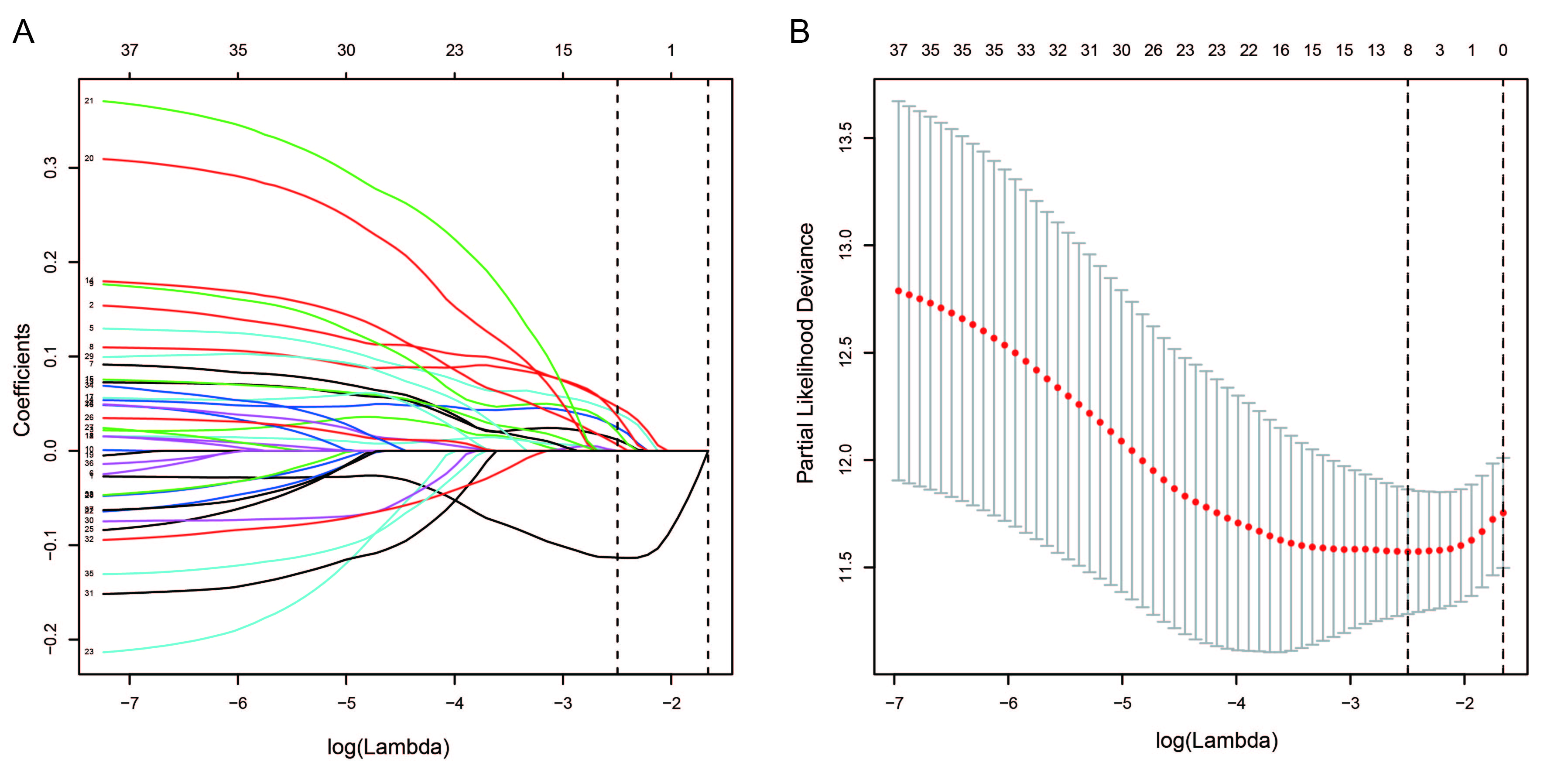

Supplement: Supplementary file 9 — FIGURE S2 [file CAM4-10-6590-s009.jpg]

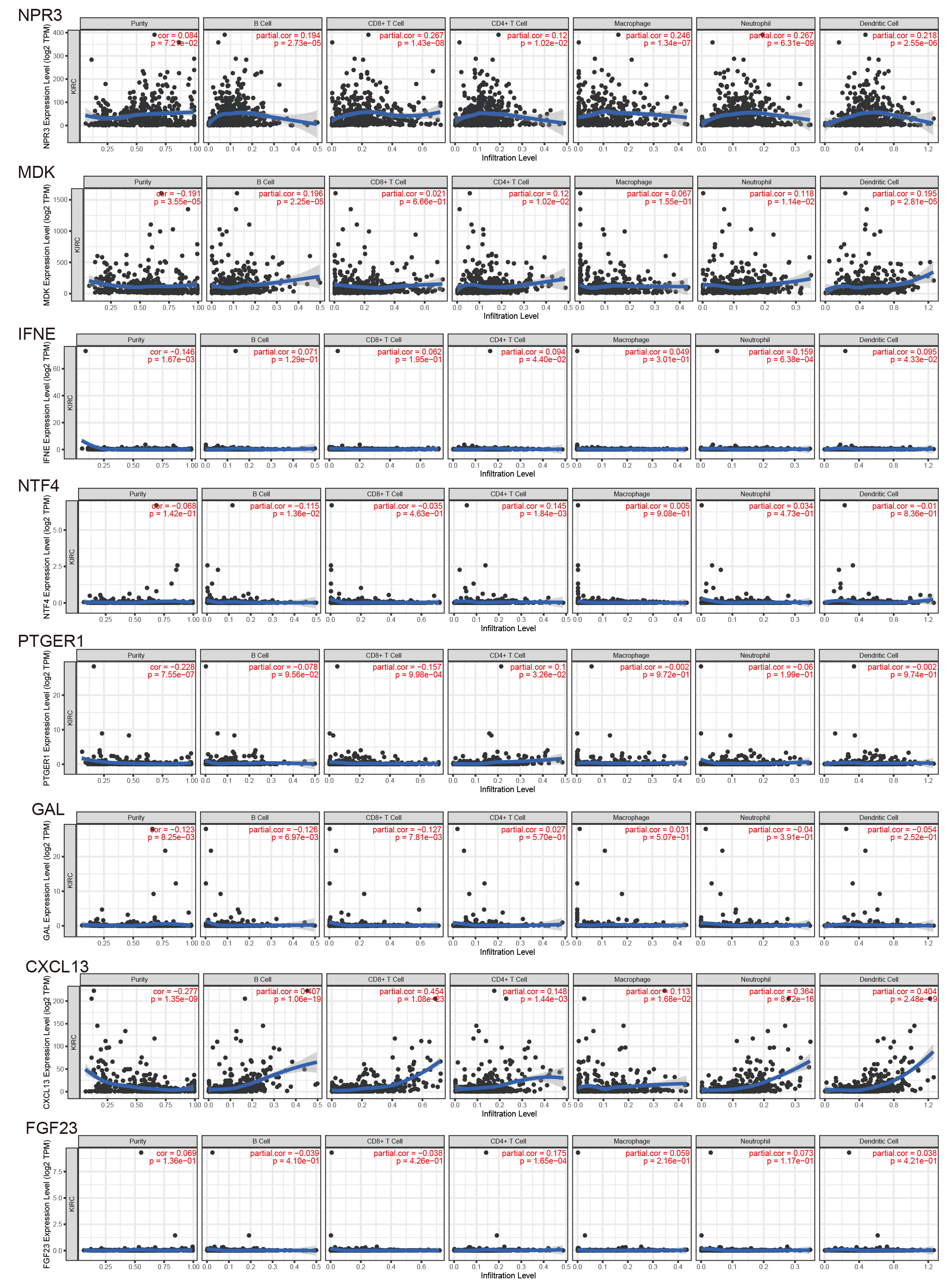

Supplement: Supplementary file 10 — FIGURE S3 [file CAM4-10-6590-s012.jpg]

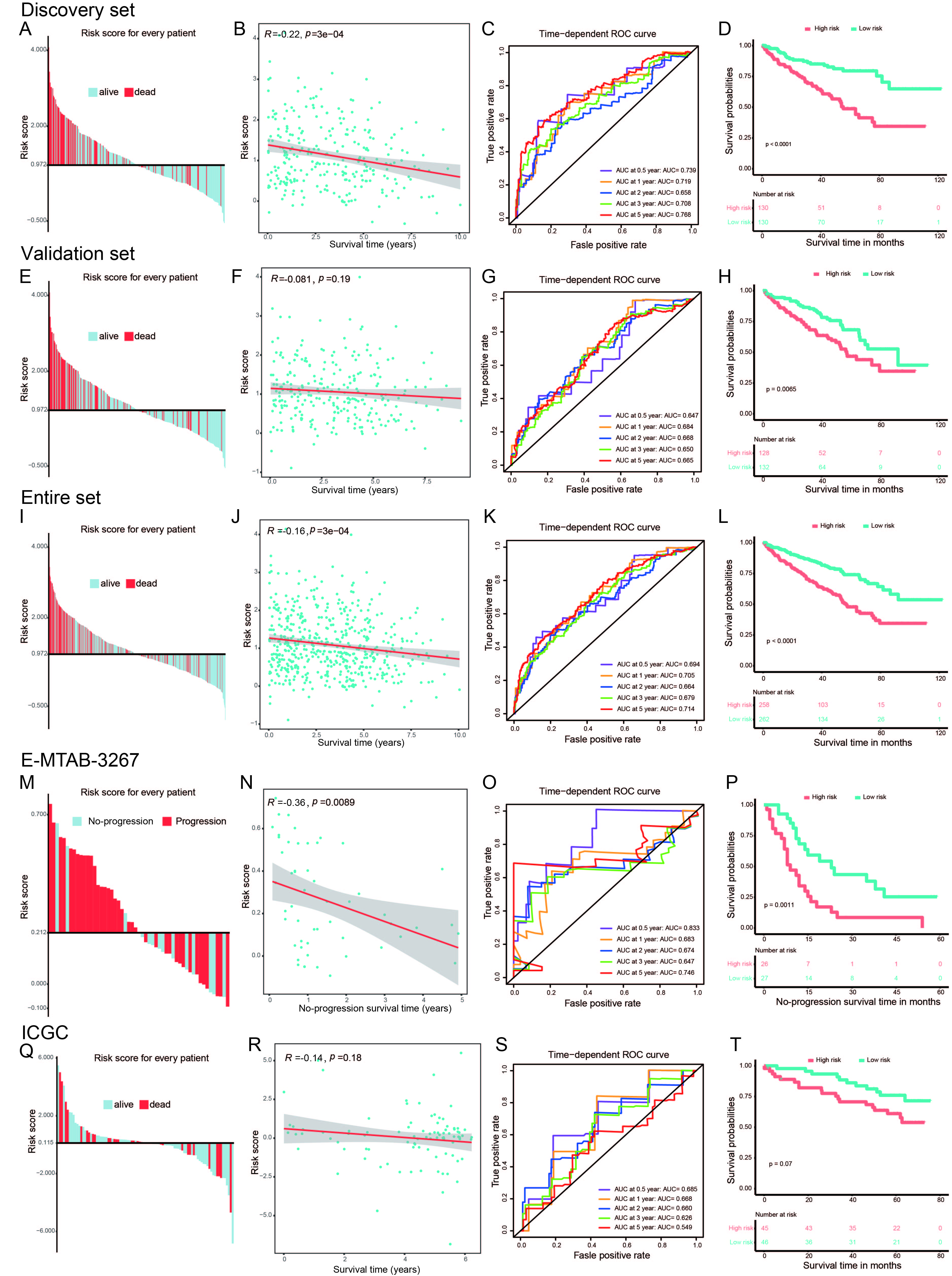

Supplement: Supplementary file 11 — FIGURE S4 [file CAM4-10-6590-s006.jpg]

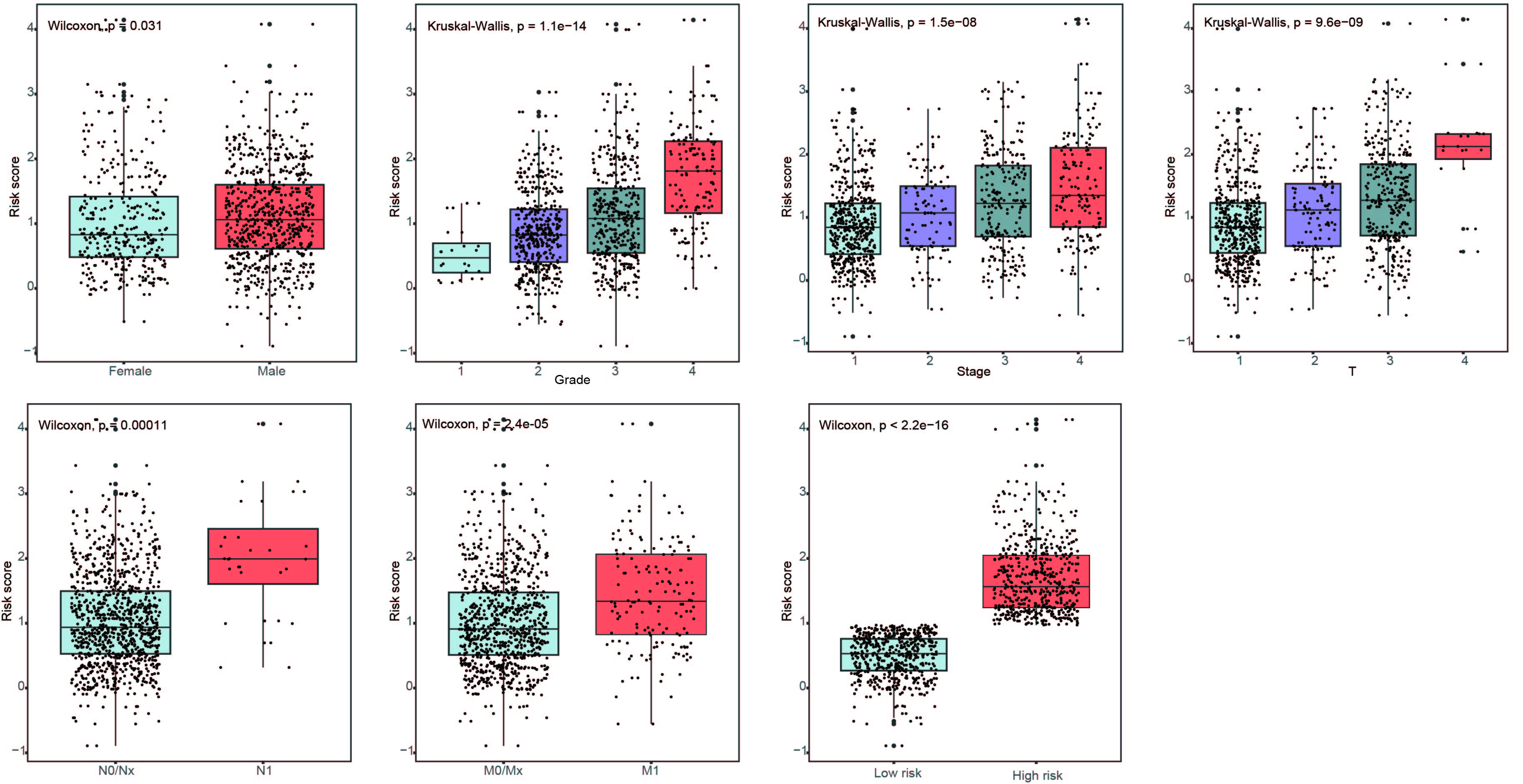

Supplement: Supplementary file 12 — FIGURE S5 [file CAM4-10-6590-s001.jpg]

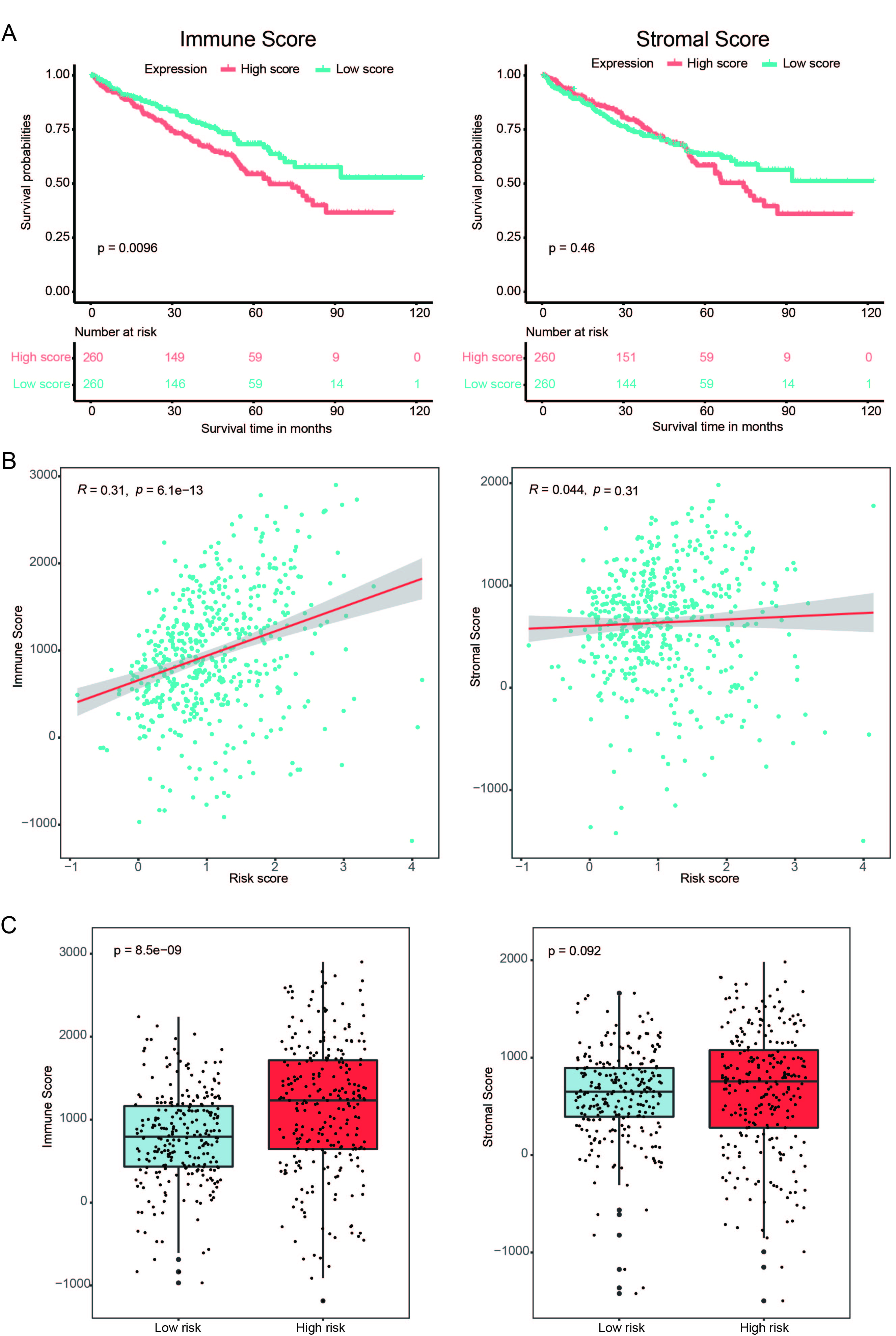

Supplement: Supplementary file 13 — FIGURE S6 [file CAM4-10-6590-s004.jpg]
